# Silencing of miR-193a-5p increases the chemosensitivity of prostate cancer cells to docetaxel

**DOI:** 10.1186/s13046-017-0649-3

**Published:** 2017-12-08

**Authors:** Zhan Yang, Jin-Suo Chen, Jin-Kun Wen, Hai-Tao Gao, Bin Zheng, Chang-Bao Qu, Kai-Long Liu, Man-Li Zhang, Jun-Fei Gu, Jing-Dong Li, Yan-Ping Zhang, Wei Li, Xiao-Lu Wang, Yong Zhang

**Affiliations:** 1 Department of Urology, The Second Hospital of Hebei Medical University, 215 Heping West Road, Shijiazhuang, 050000 China; 2 Department of Biochemistry and Molecular Biology, Ministry of Education of China, Hebei Medical University, No. 361 Zhongshan E Rd, Shijiazhuang, 050017 China; 3 Department of Science and Technology, The Second Hospital of Hebei Medical University, 215 Heping W Rd, Shijiazhuang, 050000 China; 4 Department of Emergency Medicine, The second hospital of Hebei Medical University, Shijiazhuang, Hebei 050000 China

**Keywords:** miR-193a-5p, Prostate cancer, Docetaxel, Chemoresistance, HO-1, Bach2

## Abstract

**Background:**

Docetaxel-based chemotherapy failure in advanced prostate carcinoma has partly been attributed to the resistance of prostate cancer (PC) cells to docetaxel-induced apoptosis. Hence, there is an urgent need to identify mechanisms of docetaxel chemoresistance and to develop new combination therapies.

**Methods:**

miR-193a-5p level was evaluated by qPCR in prostate tissues and cell lines, and its expression in the tissues was also examined by in situ hybridization. PC cell line (PC3 cell) was transfected with miR-193a-5p mimic or its inhibitor, and then cell apoptosis and the expression of its downstream genes *Bach2* and *HO-1* were detected by TUNEL staining and Western blotting. Luciferase reporter assay was used to detect the effect of miR-193a-5p and Bach2 on HO-1 expression. Xenograft animal model was used to test the effect of miR-193a-5p and docetaxel on PC3 xenograft growth.

**Results:**

miR-193a-5p was upregulated in PC tissues and PC cell lines, with significant suppression of PC3 cell apoptosis induced by oxidative stress. Mechanistically, miR-193a-5p suppressed the expression of *Bach2*, a repressor of the HO-1 gene, by directly targeting the *Bach2* mRNA 3′-UTR. Docetaxel treatment modestly decreased Bach2 expression and increased HO-1 level in PC3 cells, whereas a modest increase of HO-1 facilitated docetaxel-induced apoptosis. Notably, docetaxel-induced miR-193a-5p upregulation, which in turn inhibits Bach2 expression and thus relieves Bach2 repression of HO-1 expression, partly counteracted docetaxel-induced apoptosis, as evidenced by the increased Bcl-2 and decreased Bax expression. Accordingly, silencing of miR-193a-5p enhanced sensitization of PC3 cells to docetaxel-induced apoptosis. Finally, depletion of miR-193a-5p significantly reduced PC xenograft growth in vivo.

**Conclusions:**

Silencing of miR-193a-5p or blockade of the miR-193a-5p-Bach2-HO-1 pathway may be a novel therapeutic approach for castration-resistant PC.

**Electronic supplementary material:**

The online version of this article (10.1186/s13046-017-0649-3) contains supplementary material, which is available to authorized users.

## Background

Prostate cancer (PC) is one of the most common malignancies in male, and its incidence is increasing every year in the world. Organ-confined PC can be effectively treated through radical prostatectomy or radiation therapies [1]. However, for advanced prostate carcinoma, androgen deprivation therapy (ADT) is the first line of therapeutic intervention [2, 3]. Once hormone resistance develops, advanced PC is typically fatal within approximately 1 year [4]. Currently, docetaxel (Doc)-based chemotherapy is considered to be therapeutically efficacious for metastatic castration-resistant PC [5]. Unfortunately, many patients often encounter several undesirable side effects [6], and drug resistance often leads to treatment failure [7]. Thus, there is an urgent need to identify factors that influence efficacy of docetaxel therapy. Although recent studies suggest that some microRNAs (miRNAs), such as miR-375 [8], miR-200c and miR-205 [6], might be involved in docetaxel resistance of PC, the molecular mechanisms of the acquired docetaxel resistance are largely unknown.

miRNAs play a critical role in tumor progress by regulating gene expression at the posttranscriptional level. Several miRNAs, including miR-34a [9], miR-375 [8], miR-124 [10], miR-205 [11] and miR-21 [12] have been implicated in tumor drug resistance. Recent studies showed that miR-193a-5p but only suppresses tumor growth, but also promotes tumor progression through regulating cell proliferation [13, 14] and apoptosis, as well as through inducing drug resistance [15, 16]. A previous study reported that miR-193a-3p, another mature miRNA of miR-193a precursor family, regulates the multi-drug resistance of bladder cancer by targeting the LOXL4 gene [17]. However, it remains unclear whether miR-193a-5p is involved in the resistance of PC cells to docetaxel-induced apoptosis.

Heme oxygenase-1 (HO-1), a cytoprotective enzyme, exerts antioxidant, anti-inflammatory, and anti-apoptotic effect [18]. HO-1 overexpression is known to be associated with PC progression and poor clinical outcomes [19] . Under oxidative stress conditions caused by chemotherapeutic agents, cancer cells upregulate antioxidant factors, such as HO-1, and enhance their anti-apoptotic capacity to protect against oxidative injury induced by anticancer agents [20]. However, the precise mechanism underlying anticancer agent-induced HO-1 upregulation remains largely unclear.

Previous studies have demonstrated that HO-1 gene transcription is highly inducible, and its expression is regulated by the different transcription factors, such as Nrf2 [21], Bach1 [22], activator protein-1(AP-1) [23] and PPARα [24]. Furthermore, Bach2 has been shown to transcriptionally repress HO-1 expression in chronic myeloid leukemia (CML) cells, which induces apoptosis in response to oxidative stress [25]. Although low Bach2 expression was reported to be associated with high leukemic cell proliferation, unfavorable clinical features, and poor clinical outcome in acute lymphoblastic leukemia (ALL) [25, 26], there are only a few reports about the role of Bach2 in solid tumors. Moreover, the specific contribution of Bach2 to the resistance of PC cells to docetaxel-induced apoptosis has not been investigated.

In the present study, we detected the apoptosis-associated gene (Bcl-2, Bax and cleaved caspase-3) expression in human PC tissues and PC cell line in the context of docetaxel treatment. Our findings provide the evidence that regulatory crosstalk between miR-193a-5p, Bach2 and HO-1 is responsible for the resistance of PC cells to docetaxel-induced apoptosis. Furthermore, our results have linked miR-193a-5p to the regulation of Bach2 and HO-1 expression in human PC.

## Methods

### Patients

Patients (median age 65 years, range 52 to 79) underwent radical prostatectomy for localized PC (*n* = 62) and benign prostatic hyperplasia (n = 62) underwent underwent transurethral resection of the prostate (TURP) at the department of urology, the second hospital of Hebei medical university, China from July 2014 to October 2017. No treatment was administered prior to surgery. All the tissue specimens were confirmed by two experienced pathologists. Pathological grading was judged by Gleason points-scoring system. The patient characteristics are summarized in Additional file 1: Table S1. The study protocol was approved by the Ethics Committee of Second Hospital of Hebei Medical University and Verbal consent was obtained from each patient.

### Cell culture and transfection

PCa cell lines (LNCap, PC3 and DU145), bladder cancer cell lines (T24, UM-UC-3) and the human normal prostate epithelial cell line (RWPE-1) were originally obtained from the American Type Culture Collection (ATCC, Manassas, USA). The LNCap, PC3, DU145 and UM-UC-3 cells were cultured in RPMI 1640 medium (Gibco Life Technologies, Rockville, MD) containing 10% fetal bovine serum (FBS) (Foundation, Gemini, CR/US), and RWPE-1 cells were grown in K-SFM supplemented with 10% FBS; T24 cells were grown in McCoy’s 5A (Modified) Medium (Thermo Fisher, 16,600,082). All kinds of cells were incubated at 37 °C in a humidified incubator with 5% CO_2_. According to the manufacturer’s protocol, the transfection of all cells was carried out using Lipofectamine 2000 (Invitrogen). The miR-193a-5p mimics, mimic NC, miR-193a-5p inhibitors, inhibitor NC and Bach2 siRNA were purchased from GenePharma Co., Ltd. (Shanghai, China). After 24~48 h of transfection, the cells were harvested and lysed for Western blotting, and the total RNA was extracted for qRT-PCR.

### Xenograft animal model

All animal studies were approved by the Institutional Animal Care and Use Committee of Hebei Medical University (approval ID: HebMU 20,080,026), and all efforts were made to minimize suffering. Xenograft model was performed as described previously [27]. In brief, male BALB/c nude mice at 4–6 weeks of age (18–22 g) were purchased from Vital River Laboratory Animal Technology Co., Ltd. (Beijing, china). 5 × 10^6^ LV-Ctl- or LV-miR-193a-5p-infected PC3 cells were harvested by trypsinization and resuspended in 0.2 mL PBS mixed with 50% Matrigel (Collaborative Research Inc., Bedford, MA, USA); this suspension was injected subcutaneously into the right dorsal flanks. When the average volume of the tumors reached 180 mm^3^, mice were randomly divided into PBS control group or 10 mg/kg Doc group (Cayman Chemicals, Ann Arbor, MI). Mice were given intraperitoneal injection once per week for four weeks. The length and width of mouse tumor were measured twice a week with calipers. Then the following formula was used to calculate tumor volume (volume = [(length × width^2^)/2]). At the end of this experiment, the mice were euthanized by Carbon dioxide asphyxiation. At last, the tumor tissues were fixed in 4% formalin solution or flash frozen in liquid nitrogen immediately, and stored at −80 °C until further use.

### RNA extraction and quantitative real-time PCR

Clinical and xenograft tissues were homogenized with a gentle MACSTM Dissociator (Miltenyi Biotec, Bergisch Gladbach, Germany), and cultured cells were lysed using QIAzol Lysis Reagent (79306). The concentration and purity of the RNA were determined by using NanoDrop 2000 (Thomer Fisher). For microRNA, the miScripIIRT kit (QIAGEN GmbH, D-40724 Hilden, GERMANY) was used for reverse transcription, and the miScript SYBR**®** Green PCR kit was used for qRT-PCR with specific primers for miR-193a-5p, and the RNU6b (U6) was used as internal control. For large mRNA analysis, reverse transcription of RNA was performed by using the M-MLV First Strand Kit (Life Technologies). The Platinum SYBR Green qPCR Super Mix UDG Kit (Invitrogen) was used for the qRT-PCR of mRNAs. The real-time PCR experiments were carried on a CFX96™ Real-Time System (Bio-Rad). All data were normalized with GAPDH and analyzed by adopting 2^-ΔΔCt^ method as described previously [8].

### Western blot analysis

Western blotting was performed as described previously [28]. In brief, frozen tissue samples were homogenized in RIPA lysis buffer (50 mM Tris-HCl, pH 7.5, 150 mM NaCl, 1% NP-40, 0.5% Na-deoxycholate and 0.1% SDS), and cultured cells were lysed with lysis buffer (1% Triton X-100, 150 mM NaCl, 10 mM Tris-HCl, pH 7.4, 1 mM EDTA, 1 mM EGTA, pH 8.0, 0.2 mM Na_3_VO_4_, 0.2 mM phenylmethylsulfonyl fluoride, and 0.5% NP-40). Equal amounts of protein were run on 10% SDS-PAGE, and electro-transferred to a polyvinylidene fluoride (PVDF) membranes (Millipore). Membranes were blocked with 5% milk in TTBS at room temperature for 2 h and then incubated with primary antibodies overnight at 4 °C. The antibodies that were used were as follows: anti-HO-1 (1:500, ab13248), anti-Bach2 (1:500, ab83364), anti-caspase 3 (1:1000, ab13847), anti-Nrf2 (1:1000, ab31163), anti-Bcl-2 (1:1000, 12,789–1-AP), anti-Maf (1:500, 55,013–1-AP), anti-Bax (1:1000, 50,599–2-Ig) or anti-β-actin (1:1000, sc-47,778). Membranes were then incubated with the HRP-conjugated secondary antibody (1:5000, Rockland) for 1 h at room temperature. The blots were treated with the Immobilo™ Western (Millipore), and detected by ECL (enhanced chemiluminescence) Fuazon Fx (Vilber Lourmat). Images were captured and processed by FusionCapt Advance Fx5 software (Vilber Lourmat). All experiments were replicated three times.

### In situ hybridization

In situ hybridization was performed as described previously [28]. In brief, according to user manual of miRCURY LNATM microRNA ISH Optimization Kit (Exiqon), paraffin cross-sections (5-μm thick) from clinical PC tissues were deparaffinized and rehydrated for fluorescence in situ hybridization. Hybridization was performed using fluorescence-labeled miR-193a-5p probes with hybridization buffer (Exiqon) by incubation at 56 °C for 1 h in a thermo-block (Labnet, USA). After stringent washing with SSC buffer, nonspecific binding sites were blocked with 10% normal goat serum (710,027, KPL, USA). According to need, the sections were then incubated for 1 h at 37 °C with anti-HO-1 primary antibody (ab13248, Abcam) or anti-Bach2 (ab83364, Abcam) diluted 1:50 in PBS or incubated with secondary antibody directly. After washing with PBS, the sections were incubated with a rhodamine-labeled secondary antibody (031506, KPL, USA) at 37 °C for 30 min. Images were acquired by using a Leica microscope (Leica DM6000B, Switzerland) and digitized with a software of LAS V.4.4 (Leica).

### Vector construction and luciferase reporter assay

All plasmids were constructed using restriction-enzyme digestion and one-step cloning (ClonExpress II One Step Cloning Kit, C112–02; Vazyme Biotech Co., Ltd., Nanjing, PR China) or recombinant methods. The 3′ untranslated region (UTR) sequences of Bach2 containing wild-type or mutant forms of the miR-193a-5p target site were inserted into the *Xho1* and *Sal1* digested-pmir-GLO Dual-Luciferase miRNA Target Expression Vector (Promega Corp., Madison, WI, USA). 4.9 kb HO-1 promoter sequence was obtained by PCR with primer (Additional file 2: Table S3) and inserted into the *Mlu1* and *Xho1* digested-pGL3-basic vector (Promega Corp., Madison, WI, USA). Luciferase assay was performed as described previously [29]. In brief, PC3 cells were seeded into a 24-well plate, Bach2 reporter construct (wild-type or mutant) or the empty reporter vector was co-transfected with miR-193a-5p mimic and pRL-TK, or co-transfected with mimic ctl and pRL-TK, or PC3 cells were co-transfected with pGL3-HO-1-luc vector and si-Bach2. After 24 h of transfection, luciferase activity was measured using a Dual-Glo Luciferase Assay System (Promega, Madison, WI) with a Flash and Glow (LB955, Berthold Technologies) reader. The specific target activity was expressed as the relative activity ratio of firefly luciferase to Renilla luciferase.

### Immunofluorescence staining

Cells were fixed with 4% formaldehyde and pre-incubated with 10% normal goat serum (710,027, KPL, USA), and then incubated with primary antibodies anti-Bach2 (ab83364, Abcam) and anti-HO-1 (ab13248, Abcam). Secondary antibodies were fluoresce-labeled antibody to rabbit IgG (021516, KPL, USA) and rhodamine-labeled antibody to mouse IgG (031806, KPL, USA). DAPI (157,574, MB biomedical) was used for nuclear counter staining. Images were captured by confocal microscopy (DM6000 CFS, Leica) and processed by LAS AF software.

### Immunohistochemistry (IHC) analysis

Five-micrometer paraffin cross-sections of the tissues were deparaffinized in xylene solution and rehydrated by using gradient ethanol concentrations. Sections were subjected to antigen retrieval with citrate buffer. After hydrogen peroxide and protein blocking, the sections was incubated with HO-1 primary antibody at 4 °C overnight, and then was incubated in streptavidin (HRP)-biotin labeled secondary antibody. 3, 3′-diaminobenzidine was used to detect the peroxidase. Images were acquired using a Leica microscope (Leica DM6000B, Switzerland) and digitized with LAS V.4.4 (Leica). Positively stained cells were counted in at least five fields from each area with 400 × magnification.

### Chromatin immunoprecipitation (ChIP) assay

The chromatin immunoprecipitation (ChIP) assay was performed as described previously [29]. Briefly, PC3 cells were treated with docetaxel after transfected with anti-miR-ctl or anti-miR-193a-5p for 24 h. According to the manufacturer’s protocol of EZ-CHIP™ Chromatin Immunoprecipitation Kit (Millipore, #17–371), cells were crosslinked with 1% formaldehyde and sonicated to an average size of 400–600 bp. Bach2 antibody (ab83364, Abcam) and normal mouse IgG control were used for ChIP, respectively. The precipitated DNA was purified and analyzed by qRT-PCR amplification using primers specific for the HO-1 promoter.

### Cell apoptosis

TUNEL staining was performed to evaluate cell apoptosis as previously described [28]. In brief, PC3 cells were treated with 10 nM docetaxel combined with 20 μM Hemin or Znpp for 24 h and fixed by using 4% formaldehyde. Paraffin cross-sections (5-μm thick) of xenograft tissues were deparaffinized and rehydrated for TUNEL staining according to the manufacturer’s instructions (Vazyme, TUNEL Bright-Red Apoptosis Detection Kit, A113). TUNEL-positive cells were counted under fluorescence microscopy (DMI4000B, Leica).

### Target prediction

Potential target genes of miR-193a-5p were identified with following miRNA target prediction algorithms: miRanda (www.microrna.org) and RNAhybrid (http://bibiserv.techfak.uni-bielefeld.de/rnahybrid/submission.html) [30, 31].

### Statistical analysis

All of the data were represented as the means ± S.E.M. Independent Student’s *t*-test was used for comparisons of differences between two groups. The correlation between miR-193a-5p and Bach2 mRNA expression was evaluated using Spearman’s correlation analysis. Results were considered statistically significant at *p* < 0.05. Observer variation in immunohistochemical staining was analyzed by interclass correlation coefficient (ICCC) and κ statistics (κ) [32].

## Results

### miR-193a-5p is upregulated in PC tissues and PC cell lines

**Fig. 1:**
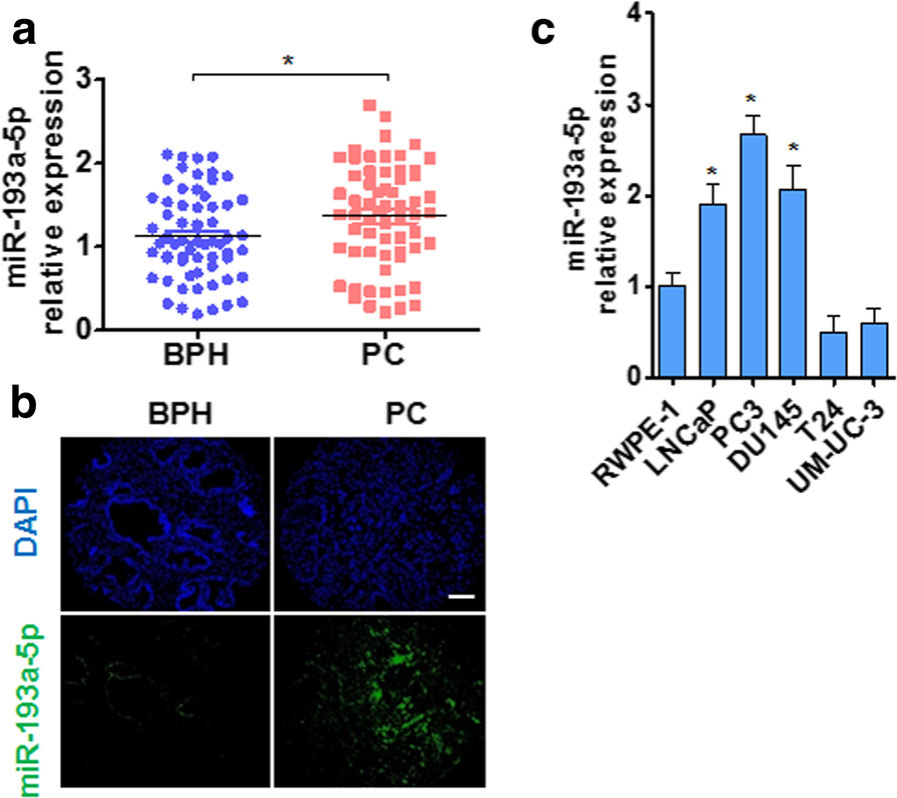
miR-193a-5p is upregulated in PC tissues and PC cell lines. **a** qRT-PCR detected the expression of miR-193a-5p in matched benign prostatic hyperplasia (BPH, *n* = 40) and PC tissues (PC, *n* = 40). *P < 0.001 vs BPH. Normalized against an internal control U6 RNA. **b** Fluorescence in situ hybridization (FISH) for detection of miR-193a-5p in PC and BPH tissues. Blue staining represents the nucleus and green staining indicates miR-193a-5p. Scale bar = 64 μm. **c** The expression levels of miR-193a-5p were detected in cancer cell lines (LNCaP, PC3, DU145, T24 and UM-UC-3) and normal prostate epithelial cells (RWPE-1); *P < 0.05 vs. RWPE-1

Because microRNA profile analysis revealed that miR-193a-5p was upregulated in human PC tissues [33], we initially used quantitative real-time PCR (qRT-PCR) to validate miR-193a-5p expression in PC tissues (Additional file 1: Table S1) and benign prostatic hyperplasia (BPH). Consistent with the microarray chip analyses, miR-193a-5p level was significantly increased in PC tissues from 62 patients compared with those from BPH patients (Fig. 1a). Further, RNA in situ hybridization in PC and BPH tissues also showed that miR-193a-5p was markedly upregulated in the PC tissues (Fig. 1b and Additional file 3: Figure S1). We also examined miR-193a-5p expression in the different PC cell lines (LNCap, PC3 and DU145) and bladder cancer cell lines (T24 and UM-UC-3) as well as in human normal prostate epithelial cell line (RWPE-1) and showed that miR-193a-5p expression was significantly increased in three PC cell lines, but not bladder cancer cell lines compared with the normal prostate epithelial cell, with miR-193a-5p level being about 1-fold up-regulated in three PC cell lines (Fig. 1c). These findings suggest that the upregulation of miR-193a-5p may be responsible for PC development.

### miR-193a-5p upregulation suppresses PC3 cell apoptosis induced by H_2_O_2_

**Fig. 2:**
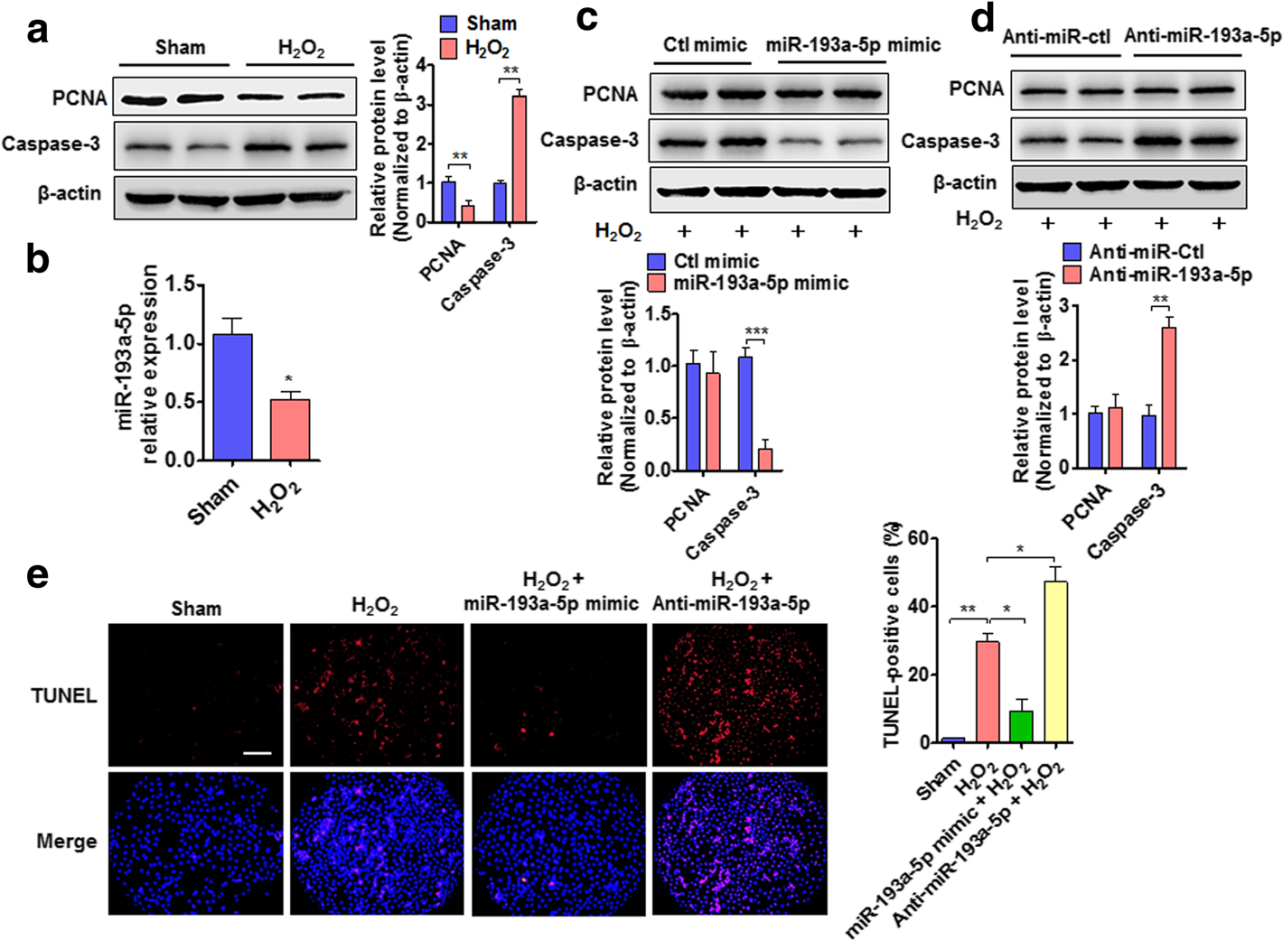
miR-193a-5p upregulation suppresses PC3 cell apoptosis. **a** Western blotting detected PCNA and cleaved caspase-3 proteins in PC3 cells treated with or without H_2_O_2_ (100 μM, 12 h). Right panel shows densitometric analysis of three independent experiments. **P < 0.01 vs. vehicle control. **b** qRT-PCR detected the expression of miR-193a-5p in PC3 cells treated with or without H_2_O_2_. *P < 0.05 vs. vehicle control. **c** Western blotting detected PCNA and cleaved caspase-3 in PC3 cells transfected with miR-193a-5p mimic or control (Ctl) mimic, and then treated with H_2_O_2_. Bottom panel shows densitometric analysis. ***P < 0.001 vs. Ctl mimic. **d** PCNA and cleaved caspase-3 were determined by western blotting in PC3 cells transfected with anti-miR-193a-5p or anti-miR-Ctl, and then treated with H_2_O_2_. Bottom panel shows densitometric analysis. **P < 0.01 vs. anti-miR-Ctl. **e** The TUNEL staining detected H_2_O_2_–induced apoptosis in PC3 cells transfected with miR-193a-5p mimic or anti-miR-193a-5p. Blue staining represents the nucleus, and red staining indicates TUNEL-positive cells. Right panel shows the number of TUNEL-positive cells of three independent experiments. Scale bar = 100 μm. *P < 0.05, **P < 0.01 vs. their corresponding control

Because oxidative stress pathway is known to be the predominant pathway affected by miR-193a-3p in bladder cancer [17], we sought to determine the effect of miR-193a-5p on oxidative stress-evoked cell proliferation and apoptosis in PC3 cells. As shown in Fig. 2a, exposure of PC3 cells to H_2_O_2_ obviously inhibited the proliferation and promoted the cell apoptosis, as shown by decreased proliferating cell nuclear antigen (PCNA) level as well as by increased cleaved caspase-3 (apoptosis marker). Simultaneously, miR-193a-5p expression was obviously downregulated in H_2_O_2_-treated PC3 cells (Fig. 2b). Next, PC3 cells were transfected with miR-193a-5p mimic or its negative control, and then treated with H_2_O_2_. The results showed that miR-193a-5p overexpression dramatically reduced the cleavage of caspase-3 induced by H_2_O_2_ treatment, but did not affect PCNA level (Fig. 2c). Conversely, when miR-193a-5p was knocked down by transfecting PC3 cells with anti-miR-193a-5p, H_2_O_2_ treatment further increased the level of cleaved caspase-3 (Fig. 2d). To provide additional confirmation that miR-193a-5p inhibits PC3 cell apoptosis, the effect of miR-193a-5p on PC3 cell apoptosis induced by H_2_O_2_ was detected by TUNEL staining. As shown in Fig. 2e, miR-193a-5p overexpression reduced, whereas knockdown of miR-193a-5p by its antagomir increased the number of TUNEL-positive cells upon PC3 cell exposure to H_2_O_2_. Collectively, these data suggest that upregulation of miR-193a-5p inhibits PC3 cell apoptosis induced by H_2_O_2_.

### miR-193a-5p mediates docetaxel regulation of HO-1 expression and suppresses PC3 cell apoptosis by increasing HO-1 expression

**Fig. 3:**
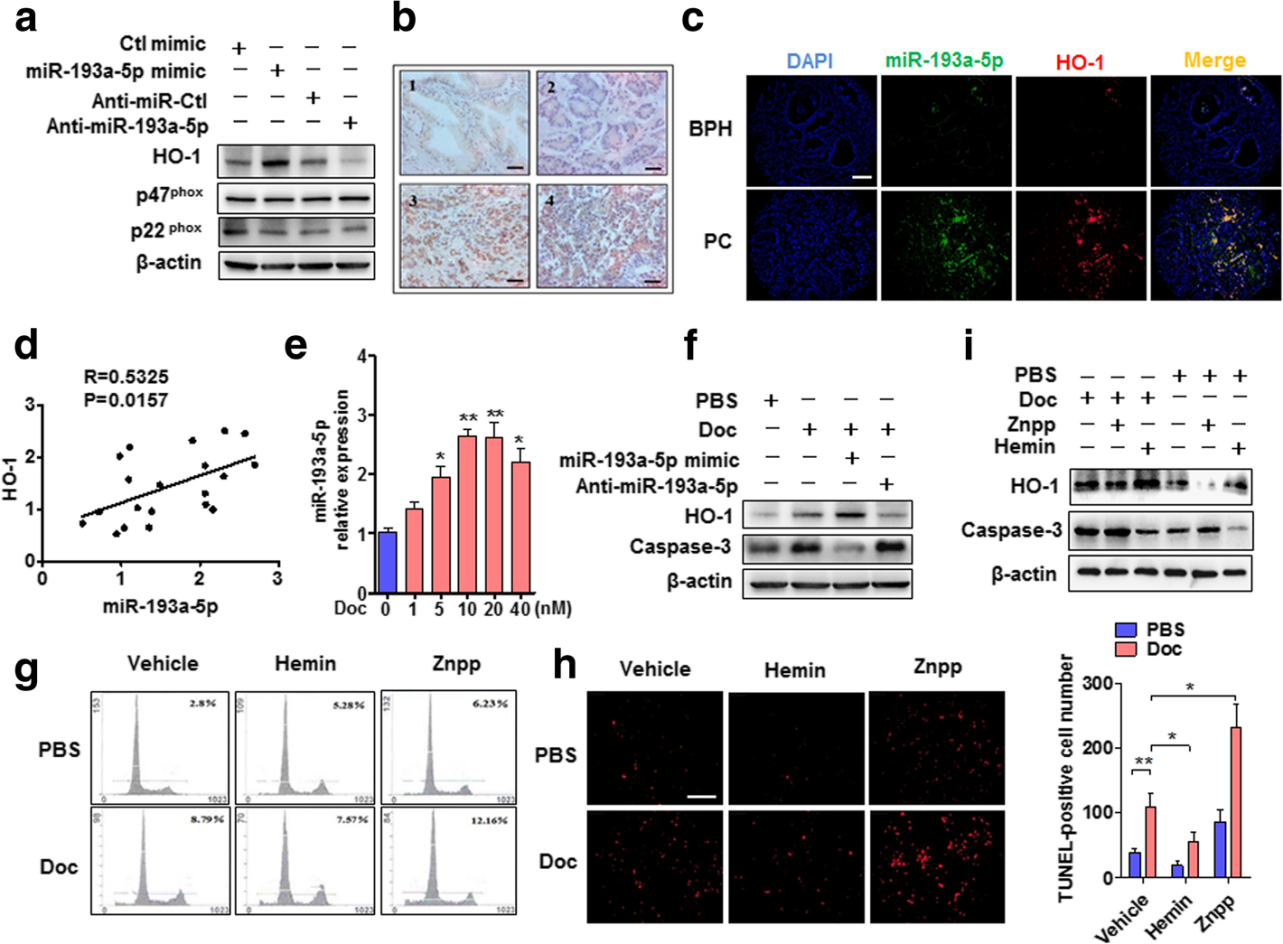
miR-193a-5p inhibits PC3 cell apoptosis by increasing HO-1 expression. **a** PC3 cells were transfected with the indicated RNA constructs and then treated with H_2_O_2_, HO-1, p47^phox^ and p22^phox^ expression was determined by Western blotting. **b** Immunohistochemical staining of HO-1 at different stages of PC progression. 1, Benign prostatic hyperplasia (BPH); 2, Gleason grade 2; 3, Gleason grade 4; 4, Gleason grade 5 PC. HO-1 expression increases with high Gleason score. Bars = 40 μm. **c** Fluorescence in situ hybridization (FISH) staining was performed on sections from BPH and PC tissues. Green, red and blue staining indicates miR-193a-5p, HO-1 and DAPI, respectively. Bar = 64 μm. **d** HO-1 mRNA and miR-193a-5p were measured by RT-qPCR, and Pearson correlation analysis shows a positive correlation between miR-193a-5p and HO-1. (*P* = 0.0157; *R* = 0.5325). **e** PC3 cells were treated with the different concentrations of Docetaxel (Doc), and the expression of miR-193a-5p was detected by qRT-PCR. *P < 0.05, **P < 0.01 vs. vehicle control. **f** Following transfection with the indicated RNA constructs, PC3 cells were treated with Doc (10 nM). HO-1 and cleaved caspase-3 were determined by Western blotting. **g** PC3 cells were treated with Doc (10 nM) alone or together with 20 μM Hemin (sigma, #51280) or Znpp (Protoporphyrin IX zinc, sigma, #282820) for 24 h, and cell apoptosis was assessed by flow cytometry. **h** PC3 cells were treated as in (G), TUNEL staining detected cell apoptosis. Right panel shows the number of TUNEL-positive cells of three independent experiments. Bar = 100 μm. *P < 0.05 vs. Doc alone; **P < 0.01 vs. vehicle control. **i** PC3 cells were treated as in (**g**), Western blotting detected HO-1 and cleaved caspase-3. Experiments were repeated three times

Because we found that miR-193a-5p inhibited PC3 cell apoptosis induced by oxidative stress, we investigated whether miR-193a-5p regulates the expression of oxidative stress-related genes, such as heme oxygenase-1 (HO-1) and NADPH oxidase subunits p47^phox^ and p22^phox^. The results showed that miR-193a-5p overexpression in PC3 cells increased, whereas antagomir-mediated silencing of miR-193a-5p reduced, the expression of HO-1, but not p47^phox^ and p22^phox^ (Fig. 3a and Additional file 4: Figure S2). In further experiments, the expression of HO-1 was determined by immunohistochemical staining in the primary PC at different stages of progression. As shown in Fig. 3b, Additional file 5: Figure S3 and Additional file 6: Table S2, there was a trend toward a positive correlation between the increased HO-1 expression and higher clinical stage (*P* = 0.035; ICCC = 0.91, κ = 0.74), higher preoperative prostate-specific antigen (PSA) levels (*P* = 0.028; ICCC = 0.89, κ = 0.81), positive seminal vesicle invasion (*P* = 0.041; ICCC = 0.86, κ = 0.72) or higher Gleason grade (*P* = 0.031; ICCC = 0.92, κ = 0.83). The results that miR-193a-5p and HO-1 were upregulated in the PC tissues prompted us to investigate whether there was a statistical correlation between HO-1 and miR-193a-5p expression levels. In situ hybridization of miR-193a-5p, combined with HO-1 immunostaining for the BPH and PC tissues, confirmed that the expression of miR-193a-5p and HO-1 was higher in the PC tissues than in BPH tissues (Fig. 3c) and that both miR-193a-5p and HO-1 were co-localizated in the PC tissues. Statistical analysis reveals a significant correlation between miR-193a-5p and HO-1 (Fig. 3d).

Considering that HO-1 and miR-193a-5p were upregulated in the PC tissues and that docetaxel is the standard of care for metastatic PC [34], we investigated the effects of docetaxel on miR-193a-5p and HO-1 expression in PC3 cells. As shown in Fig. 3e, docetaxel increased the expression of miR-193a-5p in a concentration-dependent manner, with an increase of 2.7-fold at 10 nM docetaxel. To determine whether miR-193a-5p mediates docetaxel regulation of HO-1 expression, PC3 cells were transfected with miR-193a-5p mimic or anti-miR-193a-5p, followed by treatment with docetaxel. Western blot analysis showed that docetaxel increased the levels of HO-1 and the cleaved caspase-3, and that docetaxel treatment combined with miR-193a-5p overexpression further upregulated HO-1 expression but reduced the cleavage of caspase-3, whereas depletion of miR-193a-5p by its antagomir significantly decreased docetaxel-induced HO-1 expression and promoted caspase-3 cleavage (Fig. 3f, Additional file 7: Figure S4A). To further examine whether HO-1 downregulation is responsible for docetaxel-induced PC3 cell apoptosis, PC3 cells were pretreated with Znpp (HO-1 inhibitor) or Hemin (HO-1 inducer) for 6 h, followed by treatment with docetaxel, and then analyzed by flow cytometry and TUNEL staining. As shown in Fig. 3g, docetaxel alone markedly increased PC3 cell apoptosis, with cell apoptotic rate reaching 8.79% compared to control (2.8%). HO-1 inhibitor (Znpp) combined with docetaxel increased significantly the apoptotic rate to 12.16% compared to docetaxel alone (8.79%). TUNEL staining also revealed that the combination of docetaxel with HO-1 inhibitor (Znpp) further enhanced docetaxel-induced PC3 cell apoptosis, as evidenced by the increased TUNEL-positive cell number (Fig. 3h). Consistently, docetaxel alone slightly upregulated the levels of HO-1 and the cleaved caspase-3 compared with vehicle control, while docetaxel combined with HO-1 inhibitor (Znpp) significantly suppressed HO-1 expression and increased the cleavage of caspase-3 compared with docetaxel alone. Conversely, docetaxel combined with HO-1 inducer (Hemin) markedly increased HO-1 expression and reduced PC3 cell apoptosis, as shown by the decreased caspase-3 cleavage (Fig. 3i, Additional file 7: Figure S4B). These findings indicate that miR-193a-5p may mediate docetaxel regulation of HO-1 expression, and that HO-1 upregulation induced by docetaxel increases the resistance of PC3 cells to docetaxel-induced apoptosis.

### HO-1 upregulation leads to resistance of PC3 cells to docetaxel-induced apoptosis by increasing Bcl-2 and decreasing Bax expression

**Fig. 4:**
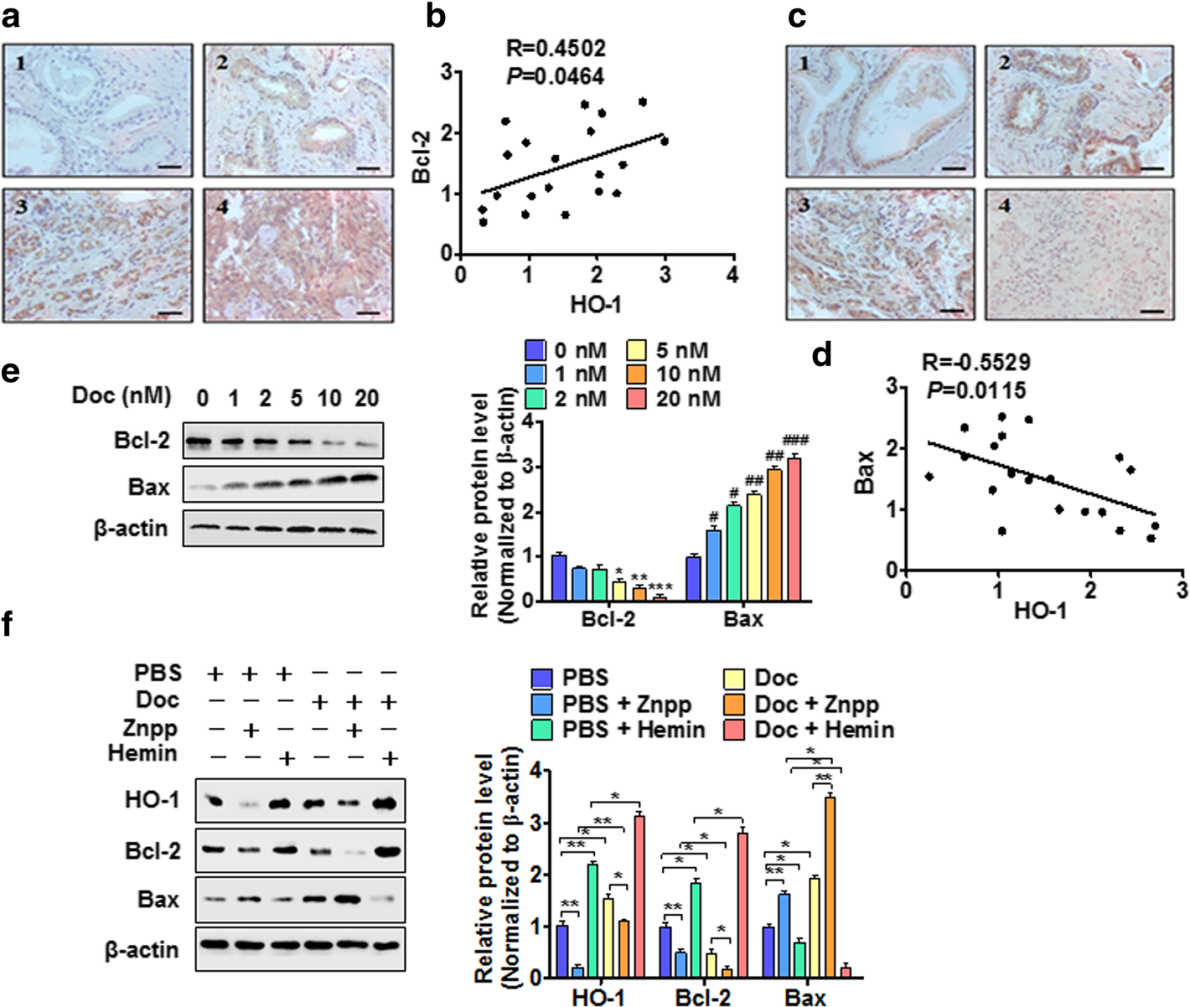
HO-1 upregulation leads to resistance of PC3 cells to docetaxel-induced apoptosis via the Bcl2/Bax pathway. **a** and **c** Immunohistochemical staining of Bcl-2 (**a**) and Bax (**c**) at different stages of PC progression. 1, BPH; 2, Gleason grade 2; 3, Gleason grade 4; 4, Gleason grade 5 PC. Bars = 40 μm. **b** and **d** Bcl-2, HO-1 and Bax mRNAs were measured by qRT-PCR, the correlation between Bcl-2 and HO-1 or between Bax and HO-1 was evaluated by Pearson correlation analysis, respectively. (**b**, *P* < 0.05, *R* = 0.4502; (**d**), *P* < 0.05, *R* = −0.5529). **e** PC3 cells were treated with the different concentrations of Doc, and Western blot analysis detected Bcl-2 and Bax expression. Right panel shows densitometric analysis of three independent experiments. *P < 0.05, **P < 0.01, ***P < 0.001 vs. vehicle control; ^#^ *P* < 0.05, ^##^ *P* < 0.01, ^###^ *P* < 0.001 vs. vehicle control. **f** PC3 cells were treated with Doc alone or together with Hemin or Znpp for 24 h, HO-1, Bcl-2 and Bax were determined by Western blot analysis. Right panel shows densitometric analysis of three independent experiments. *P < 0.05, **P < 0.01 vs. their corresponding control

To clarify the mechanism whereby HO-1 upregulation leads to resistance of PC3 cells to docetaxel-induced apoptosis, we determined the relationship between HO-1 upregulation and the expression of apoptosis-related genes Bcl-2 and Bax. Immunohistochemical staining of PC tissues showed that the expression of anti-apoptotic gene Bcl-2 markedly increased in PC tissues compared with BPH tissues, and that high Bcl-2 expression was significantly associated with high Gleason grade (*P* = 0.02; ICCC = 0.93, κ = 0.88) (Fig. 4a, Additional file 8: Figure S5A). Moreover, a stronger correlation was observed between Bcl-2 and HO-1 mRNAs (Pearson correlation, *R* = 0.4502; *P* = 0.0464; Fig. 4b, Additional file 8: Figure S5B). Reversely, the expression of pro-apoptotic gene Bax was downregulated in PC tissues, and there was a negative correlation between Bax and HO-1 mRNAs (Pearson correlation, *R* = −0.5529; *P* = 0.0115; Fig. 4d; ICCC = 0.89, κ = 0.83). Next, we treated PC3 cells with different concentrations of docetaxel and measured the effects of docetaxel on Bcl-2 and Bax expression. As shown in Fig. 4e, docetaxel significantly decreased Bcl-2 protein level and increased Bax expression in a dose-dependent manner. Notably, docetaxel combined with HO-1 inhibitor (Znpp) markedly repressed Bcl-2 expression and enhanced Bax expression compared with docetaxel alone, whereas docetaxel, in combination with HO-1 inducer (Hemin), robustly increased Bcl-2 and reduced Bax expression level (Fig. 4f). These findings suggest that HO-1 upregulation leads to resistance of PC3 cells to docetaxel-induced apoptosis by increasing Bcl-2 and decreasing Bax expression.

### Bach2 is a direct target of miR-193a-5p

**Fig. 5:**
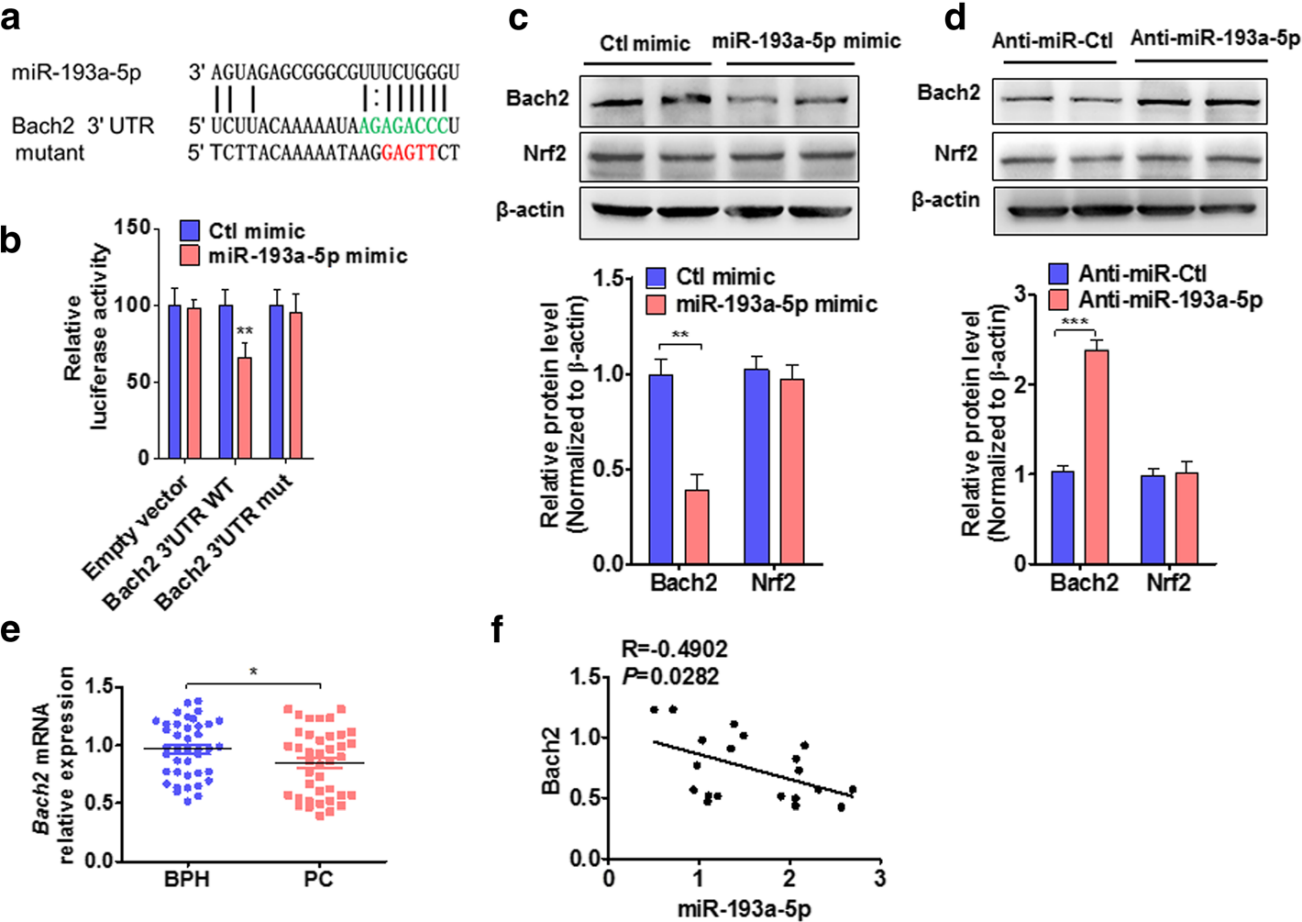
Bach2 is a direct target of miR-193a-5p. **a** Prediction of miR-193a-5p binding site at Bach2 3′-UTR. Red color indicates the sequence of the mutated miR-193a-5p binding site. **b** Luciferase reporter assays were performed in PC3 cells co-transfected cells with miR-193a-5p mimic and wild-type (WT) or mutant (mut) Bach2 3′-UTR-luciferase reporter **P < 0.01 vs. Ctl mimic. **c** and **d** PC3 cells were transfected with miR-193a-5p mimic (**c**) or anti-miR-193a-5p (**d**), Bach2 and Nrf2 expression was analyzed by Western blotting. Bottom panel shows densitometric analysis of three independent experiments. **P < 0.01, ***P < 0.001 vs. their corresponding control. **e** Bach2 mRNA was detected by qRT-PCR in BPH (*n* = 21) and PC tissues (n = 21). *P < 0.01 vs. BPH. **f** Pearson correlation was used to analyze the relationships between miR-193a-5p and Bach2. (*R* = −0.4902, *P* = 0.0282)

We next determined the mechanism whereby miR-193a-5p regulates HO-1 expression. Because previous study showed that transcription factor Bach2 served as a repressor and Nrf2 as an enhancer of the HO-1 gene [29, 35], we investigated the effects of miR-193a-5p and docetaxel on Bach2 and Nrf2 expression. Using a qRT-PCR analysis, we showed that transfecting PC3 cells with miR-193a-5p mimic alone or combined with docetaxel treatment significantly attenuated the expression of Bach2, but not Nrf2, Bach1 or Hif1α (Additional file 9: Figure S6). Then, we used a bioinformatics approach to search for the potential matching site of miR-193a-5p in the Bach2 3′-UTR and found that Bach2 3′-UTR contains a putative miR-193a-5p binding site (Fig. 5a). To confirm whether Bach2 is a direct target of miR-193a-5p, we co-transfected cells with miR-193a-5p mimic and wild-type (WT) or mutant (mut) Bach2 3′-UTR-luciferase reporter and showed that miR-193a-5p mimic significantly decreased luciferase activity mediated by wild-type Bach2 3′-UTR by 65% compared to the control (*P* < 0.01); mutation of the miR-193a-5p-binding site almost completely restored luciferase activity in the presence of the miR-193a-5p mimic (Fig. 5b). Further, PC3 cells were transfected with miR-193a-5p mimic or its antagomir, respectively, and Bach2 and Nrf2 expressions were detected by Western blotting. The results showed that miR-193a-5p mimic significantly reduced the protein level of Bach2 but not Nrf2 (Fig. 5c), while miR-193a-5p depletion by its antagomir markedly increased the level of Bach2 but not Nrf2 (Fig. 5d). Taken together, these data suggest that miR-193a-5p downregulates Bach2 expression by directly targeting the miR-193a-5p-binding site in the Bach2 3′-UTR.

In further experiments, we detected Bach2 expression in PC and BPH tissues by using qRT-PCR and found that Bach2 mRNA levels were significantly lower in PC tissues than in BPH (Fig. 5e). Moreover, the increased levels of miR-193a-5p correlated with the decreased expression of Bach2 in human PC tissues (Fig. 5f).

### Silencing of miR-193a-5p enhances sensitization of PC3 cells to docetaxel-induced apoptosis through upregulating Bach2 expression

**Fig. 6:**
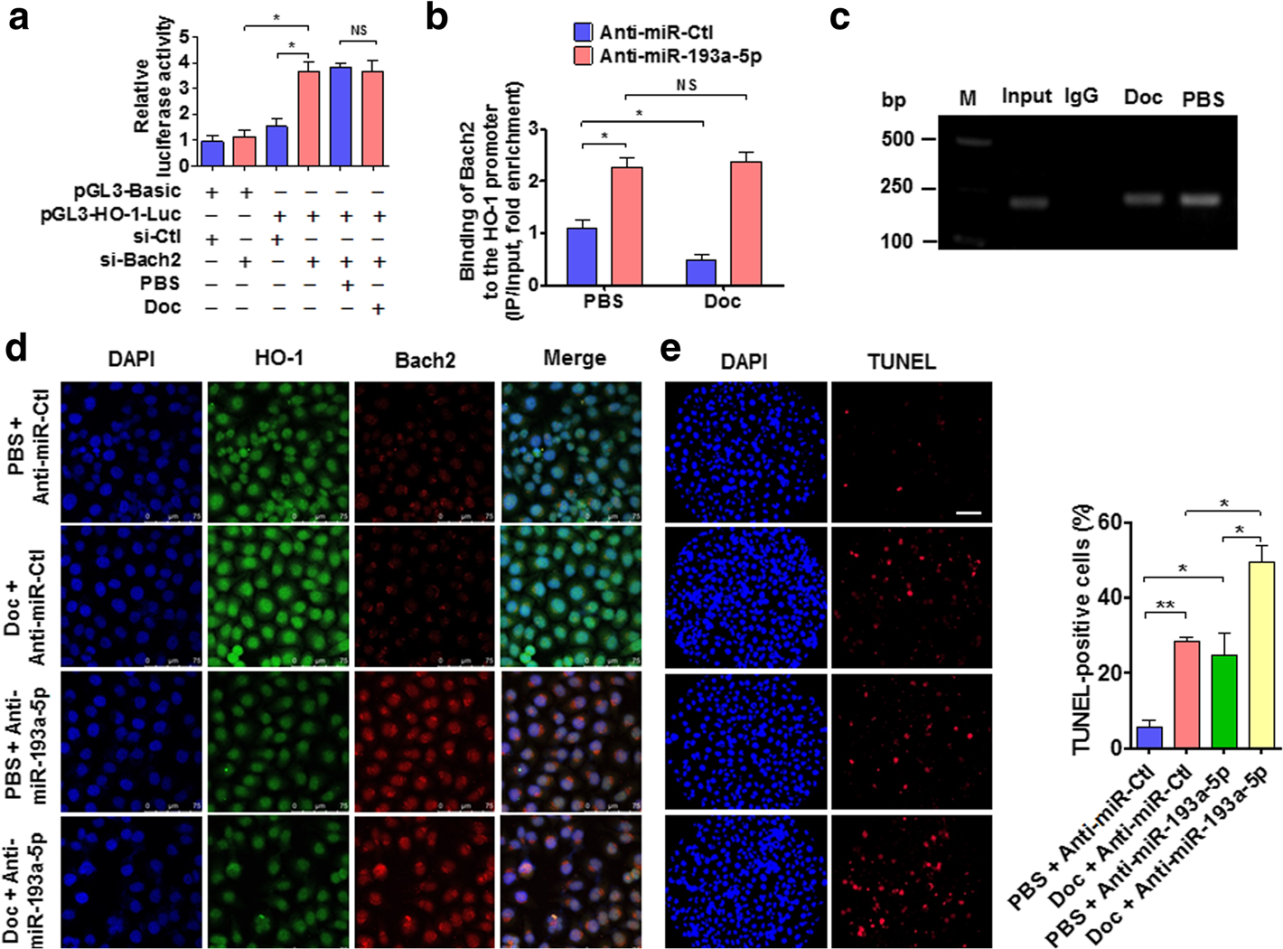
Silencing of miR-193a-5p enhances sensitization of PC3 cells to docetaxel-induced apoptosis through upregulating Bach2 expression**. a** PC3 cells were co-transfected with HO-1 promoter reporter construct and si-Bach2, and then treated with or without Doc. Luciferase reporter assays were performed. *P < 0.01 vs. empty vector or si-Ctl. **b** ChIP-qPCR detected Bach2 binding to the HO-1 promoter in PC3 cells transfected with anti-miR-193a-5p or anti-miR-Ctl, and then treated with or without Doc. *P < 0.05 vs. anti-miR-Ctl plus PBS. **c** Agarose gel electrophoresis for PCR products of chromatin immunoprecipitation showing Bach2 binding to the HO-1 promoter. **d** PC3 cells were transfected with anti-miR-193a-5p or anti-miR-Ctl, and then treated with or without Doc. Immunofluorescence staining detected the expression of HO-1 (green) and Bach2 (red). Nuclei were staining with DAPI (blue). Scale bars = 75 μm. **e** PC3 cells were treated as in (**d**), cell apoptosis was assessed by TUNEL staining. Right panel shows the number of TUNEL-positive cells of three independent experiments. *P < 0.01, **P < 0.01 vs. their corresponding control

Because Bach2 is known to act as a proapoptotic factor by repressing the antiapoptotic factor HO-1 expression [25], and because the increased HO-1 and miR-193a-5p and the decreased Bach2 were found in PC tissues, we sought to investigate the relationship between HO-1, miR-193a-5p and Bach2. First, we used a luciferase reporter assay to detect whether docetaxel affects Bach2 regulation of HO-1 expression in PC3 cells. As shown in Fig. 6a, knockdown of Bach2 significantly increased the HO-1 promoter-driven luciferase activity. Moreover, in Bach2-knocked down PC3 cells, docetaxel treatment did not affect the HO-1 promoter reporter activity. To further examine the effects of miR-193a-5p and docetaxel on Bach2 expression, ChIP-qPCR was performed. The results showed that Bach2 was recruited to Maf recognition elements (MARE) of the HO-1 promoter region, possibly through forming homodimers or heterodimers with Maf-related transcription factors [36], and that depletion of miR-193a-5p by its antagomir increased Bach2 binding to this site by approximately 2-fold (Fig. 6b). However, depletion or overexpression of miR-193a-5p did not affect the expression of Maf (Additional file 10: Figure S7). Importantly, docetaxel treatment dramatically reduced the binding of Bach2 to the HO-1 promoter in anti-miR-Ctl-transfected cells but not in miR-193a-5p-knocked down cells (Fig. 6b), implying that docetaxel-upregulated miR-193a-5p (Fig. 3e) suppresses Bach2 expression, thus leading to a decreased binding of Bach2 to the HO-1 promoter. Consistently, the agarose gel electrophoresis for the PCR products of the ChIP also showed that Bach2 binding to the HO-1 promoter was markedly decreased in docetaxel-treated PC3 cells compared with PBS-treated cells (Fig. 6c).

Using immunofluorescence staining, we further determined the effects of docetaxel treatment and miR-193a-5p knockdown on Bach2 and HO-1 expression. We found that docetaxel slightly decreased Bach2 expression, especially in the nucleus, and increased HO-1 level in anti-miR-Ctl-transfected cells, indicating that a modest increase of HO-1 level facilitates docetaxel-induced cell apoptosis. However, in miR-193a-5p-depleted PC3 cells, docetaxel markedly elevated Bach2 levels in the nucleus and attenuated HO-1 expression compared with the corresponding control group (Fig. 6d). Accordingly, when miR-193a-5p-depleted PC3 cells were treated with docetaxel, TUNEL-positive cells were significantly increased compared with docetaxel alone or miR-193a-5p knockdown alone (Fig. 6e). Together, these results suggest that docetaxel-induced miR-193a-5p upregulation, which in turn inhibits Bach2 expression and thus relieves Bach2 repression of HO-1 expression, partly counteracts docetaxel-induced apoptosis, and that silencing of miR-193a-5p enhances sensitization of PC3 cells to docetaxel-induced apoptosis.

### miR-193a-5p, Bach2 and HO-1 constitute a regulatory axis and coordinate docetaxel-induced apoptosis in PC3 cells

**Fig. 7:**
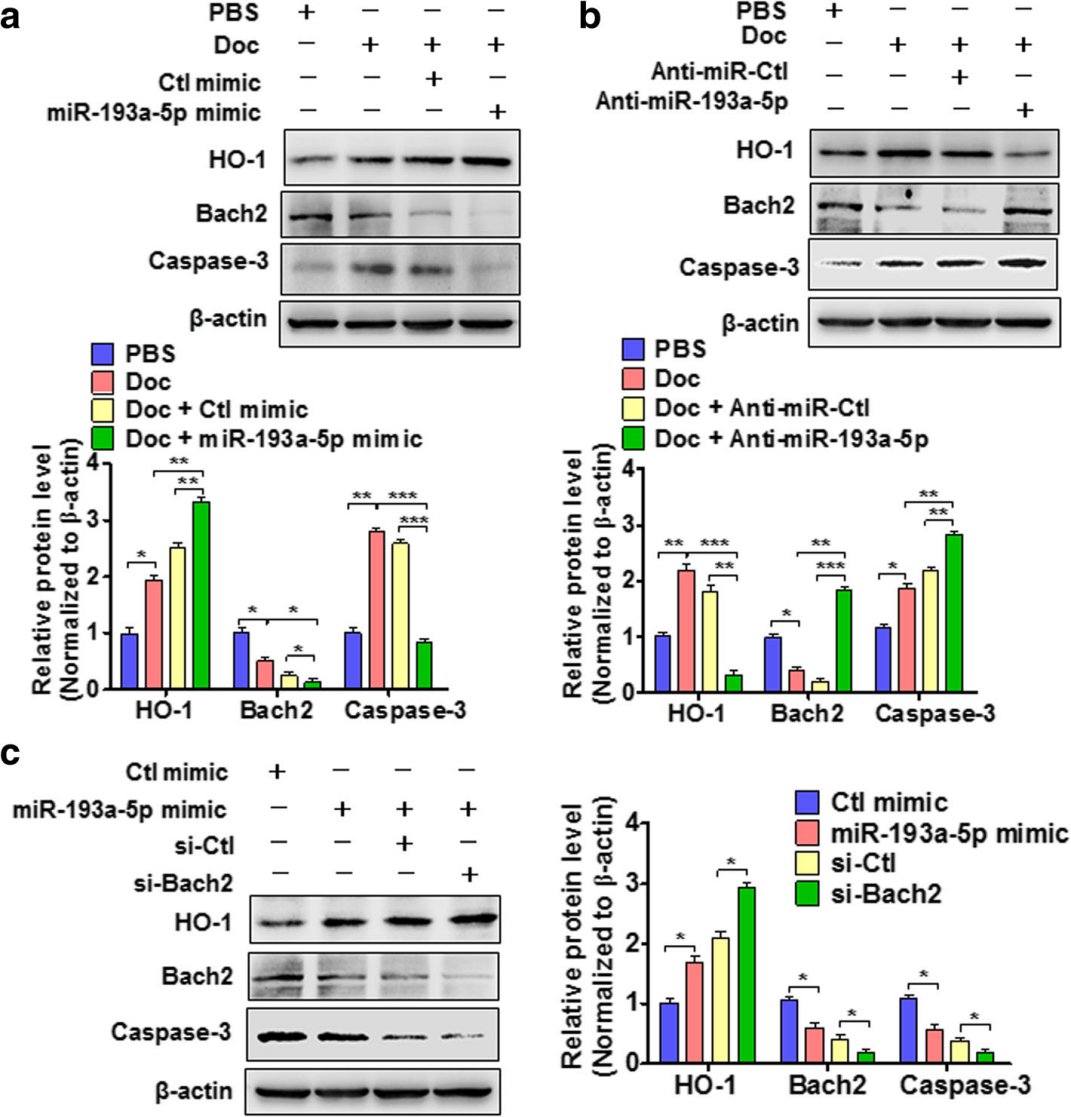
miR-193a-5p, Bach2 and HO-1 coordinate docetaxel-induced apoptosis in PC3 cells. **a** PC3 cells were transfected with miR-193a-5p mimic or Ctl mimic and then treated with or without Doc, and HO-1, Bach2 and cleaved caspase-3 were detected by Western blotting. Bottom panel shows densitometric analysis of three independent experiments. *P < 0.01, **P < 0.01, ***P < 0.001 vs. their corresponding control. **b** Western blotting detected HO-1, Bach2 and cleaved caspase-3 in PC3 cells transfected with anti-miR-193a-5p or Anti-miR-Ctl and then treated with or without Doc. Bottom panel shows densitometric analysis of three independent experiments. *P < 0.01, **P < 0.01, ***P < 0.001 vs. their corresponding control. **c** Western blotting detected HO-1, Bach2 and cleaved caspase-3 in PC3 cells transfected with the indicated RNA constructs. Right panel shows densitometric analysis of three independent experiments. *P < 0.01 vs. their corresponding control

To further clarify the role of miR-193a-5p, Bach2 and HO-1 in docetaxel-induced PC3 cell apoptosis, gain- and loss-of-function experiments of miR-193a-5p and Bach2 were performed. As shown in Fig. 7a, docetaxel treatment reduced Bach2 expression and increased HO-1 level to some extent,accompanied by an increase in the cleaved caspase-3. When PC3 cells were transfected with miR-193a-5p mimic, docetaxel sharply reduced Bach2 level and enhanced HO-1 expression, accompanied by significant inhibition of cell apoptosis, as shown by the decreased expression of cleaved caspase-3. On the contrary, when miR-193a-5p was depleted by its antagomir, docetaxel markedly increased Bach2 level and attenuated HO-1 expression, with a significant increase in the cleaved caspase-3 compared with docetaxel treatment alone (Fig. 7b), suggesting that miR-193a-5p mediates the resistance of PC3 cells to docetaxel-induced apoptosis by downregulating Bach2 and thus increasing HO-1 expression. In further experiments, we examined whether Bach2 knockdown influences the effects of miR-193a-5p on cell apoptosis and found that, in Bach2-knocked down cells, miR-193a-5p overexpression further enhanced HO-1 expression, which is accompanied by a decrease in the cleaved caspase-3 (Fig. 7c). These results indicate that miR-193a-5p, Bach2 and HO-1 constitute a regulatory axis and cooperatively regulate docetaxel-induced apoptosis in PC3 cells.

### Depletion of miR-193a-5p reduces PC xenograft growth in vivo

**Fig. 8:**
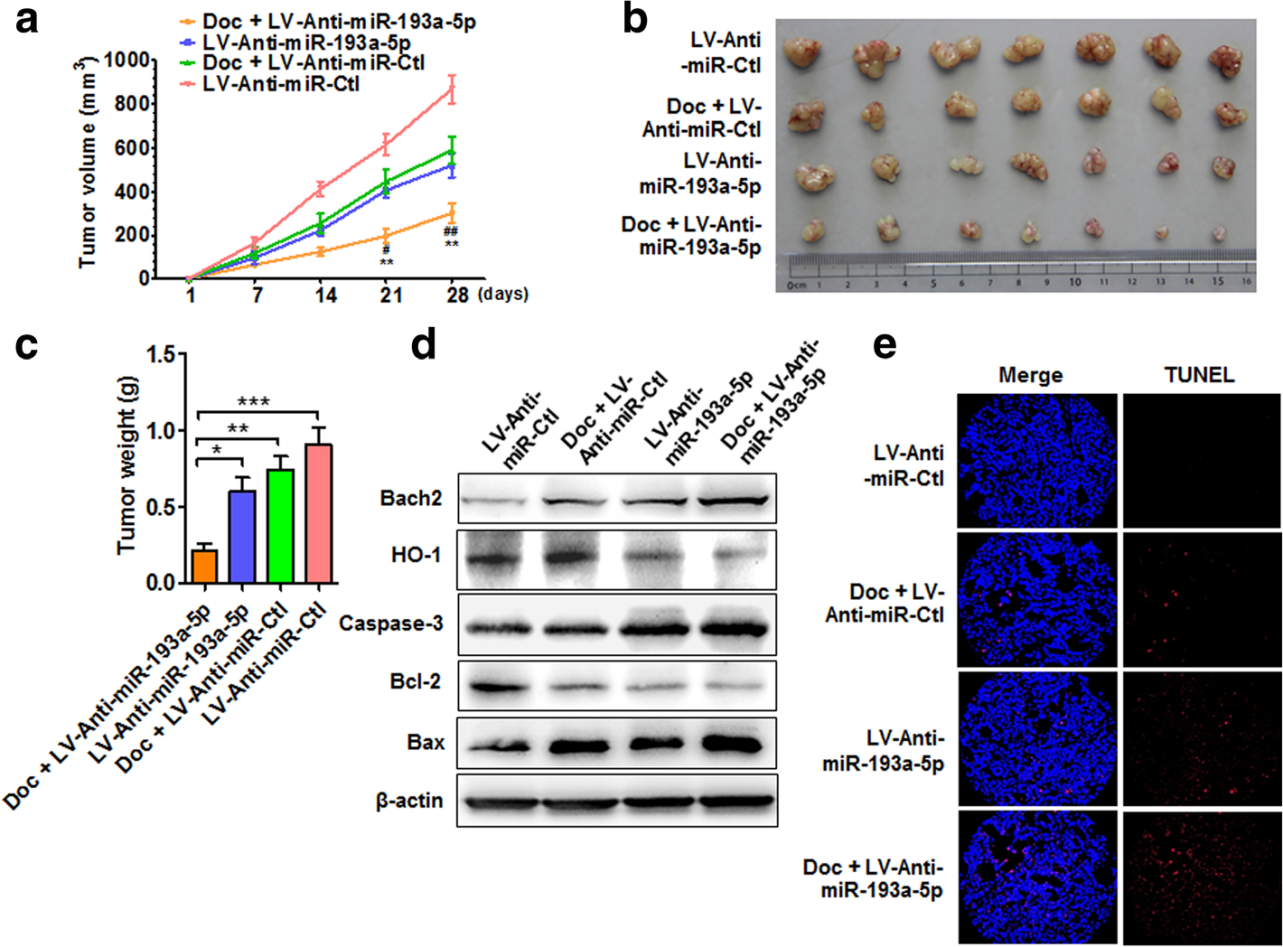
Depletion of miR-193a-5p reduces PC xenograft growth in vivo. **a** PC3 cells engineered to stably express anti-miR-193a-5p (LV-anti-miR-193a-5p) or negative control (LV-miR-Ctl) were injected subcutaneously in 200 μl PBS/Matrigel (50: 50) into the right forelimb to establish xenograft tumors. From the first day, mice were intraperitoneally injected with 10 mg/kg Doc every three days. Tumor volumes were monitored by direct measurement with calipers and calculated by the formula: (length × width^2^)/2. **P < 0.01 vs. Doc + LV-miR-Ctl; ^#^ *P* < 0.05, ^##^ *P* < 0.01 vs. LV-anti-miR-193a-5p (each groups, *n* = 16). **b** Representative tumor sizes in each group of mice. **c** Xenograft tumor wet weight in each group of mice. *P < 0.05, **P < 0.01, ***P < 0.001 vs. Doc + LV-anti-miR-193a-5p. **d** The xenograft models of nude mice were prepared as in (A), Western blotting detected the expression of Bach2, HO-1, cleaved caspase-3, Bcl-2 and Bax in xenograft tumors. **e** The xenograft models of nude mice were prepared as in (**a**), the TUNEL staining detected cell apoptosis in xenograft tumors. Blue staining represents the nucleus, and red staining indicates TUNEL-positive cells

Next, using a nude mouse xenograft model, we further corroborated the above findings that miR-193a-5p knockdown enhanced the chemosensitivity of PC3 cells to docetaxel. To test this, we implanted the PC3 cells stably expressing anti-miR-193a-5p (miR-193a-5p inhibitor) into nude mice, and observed the effects of docetaxel on PC3 xenograft growth. As expected, tumor volumes were significantly decreased in nude mice treated with docetaxel alone as well as in miR-193a-5p-depleted mice compared with vehicle control; docetaxel treatment combined with miR-193a-5p-depletion further reduced tumor volumes (Fig. 8a and b). Simultaneously, the mean wet weight of tumors was significantly lower in nude mice treated with docetaxel, in combination with miR-193a-5p-depletion, than in mice treated with docetaxel alone (Fig. 8c). Clearly, the depletion of miR-193a-5p suppressed xenograft PC3 tumor growth in nude mice. Further, we detected the expression of apoptosis-related genes Bcl-2 and Bax in xenograft tumors. The results showed that docetaxel combined with miR-193a-5p-depletion significantly upregulated the levels of Bach2, cleaved caspase-3 and Bax, accompanied by a decrease in HO-1 and Bcl-2 expression compared with vehicle control (Fig. 8d and Additional file 11: Figure S8). We also used TUNEL staining to detect cell apoptosis in xenograft tumors and found that miR-193a-5p-depletion remarkably facilitated docetaxel-induced apoptosis (Fig. 8e and Additional file 12: Figure S9). These data again suggest that miR-193a-5p knockdown increases the sensitization of PC cells to docetaxel-induced apoptosis.

## Discussion

In this study, we found that 1) miR-193a-5p is upregulated in PC tissues and cell lines, 2) miR-193a-5p promotes HO-1 expression through downregulating Bach2 level, 3) HO-1 upregulation leads to resistance of PC3 cells to docetaxel-induced apoptosis, 4) miR-193a-5p, Bach2 and HO-1 constitute a regulatory axis and coordinate docetaxel-induced apoptosis in PC3 cells, and 5) Silencing of miR-193a-5p enhances sensitization of PC3 cells to docetaxel-induced apoptosis and reduces PC xenograft growth in vivo.

Previous studies have demonstrated that STAT1 [37], PIM-1 [38], and β3-tubulin [39] might be implicated in docetaxel resistance of PC. Mechanistically, STAT1 mediates the resistance of DU145 cells to docetaxel through inducing the expression of clusterin that is involved in cell survival in the presence of docetaxel, blockage of STAT1 expression by siRNA decreases clusterin expression and inhibits PC cell proliferation by re-sensitizing drug-resistant tumor cells to docetaxel [38]. Serine/threonine kinase PIM-1 protects PC cells from apoptosis induced by docetaxel through phosphorylating transmembrane drug efflux pump BCRP/ABCG2 [39]. Functional overexpression or knockdown of β3-tubulin modulates PC cell line sensitivity to docetaxel presumably through altering cell morphology and the rate of cell proliferation [40].

In the recent decades, several microRNAs have been identified to be involved in tumor development and progression through acting either as tumor suppressors or oncogenes [40]. For example, a previous study reported that the expression of miR-200 family members miR-200c and miR-205, which function as key regulators of EMT, was significantly reduced in docetaxel-resistant cells [6]. Transfection of miR-200c and miR-205 restored E-cadherin expression level, accompanied by increased apoptosis, in docetaxel-resistant cells, suggesting that reduced miR-200c and miR-205 levels during chemotherapy are responsible for cancer cell survival and drug resistance [6]. Our previous study showed that miR-146a functioned as a tumor suppressor in PC cells, and increased miR-146a expression in both LNCaP and PC3 cells by 5-Aza-2′-deoxycytidine correlated with delayed progression of castration-resistant PC [27]. In some studies, miR-193a-5p was downregulated in several types of cancers, therefore, miR-193a-5p is believed to be an important tumor inhibitor. However, miR-193a-5p was also reported to be upregulated in certain cancer types including PC [33, 41]. Thus, miR-193a-5p could play a dual role in tumor development and progression, depending on the type of cancers or anticancer drugs used in cancer therapy. In this study, we found that miR-193a-5p was upregulated in PC tissues and PC cell lines, and the upregulation of miR-193a-5p was closely associated with PC development. We further confirmed that miR-193a-5p expression was significantly decreased in H_2_O_2_-treated PC3 cells, and miR-193a-5p mimic reduced, whereas its antagomir increased PC3 cell apoptosis induced by oxidative stress. Importantly, miR-193a-5p upregulation also attenuated PC3 cell apoptosis induced by docetaxel, while depletion of miR-193a-5p enhanced sensitization of PC cells to docetaxel-induced apoptosis.

Apoptosis is a physiological process that eliminates abnormal or nonfunctional cells and is critical for maintenance of tissue homeostasis, and failure of apoptosis results in accumulation of abnormal cells, potentially leading to tumor development [42]. Cell apoptosis is regulated at multiple levels and involves anti-apoptotic protein Bcl-2 and pro-apoptotic protein Bax. Various chemotherapeutic drugs have been shown to induce apoptosis in both in vitro and in vivo studies, suggesting that apoptosis plays a crucial role in cancer treatment [43]. It is well known that the ability of docetaxel to kill tumor cells depends partly on its ability to induce apoptosis in tumor cells [7]. HO-1 has an anti-apoptotic effect on certain cancer cells by regulating cellular homeostasis and promoting cell survival [44], these effects would be relevant to resistance to chemotherapy [45]. Indeed, HO-1 upregulation was observed in different human cancers [46], and HO-1 expression level was closely related to the disease severity of cancers. Sacca et al. revealed that the degree of HO-1 expression in the nuclei of PC cells was positively correlated with Gleason score, the higher the Gleason score, the more the number of nuclear HO-1-positive staining [47]. These results clearly suggest that HO-1 upregulation facilitates the progression of cancers. Consistent with these reports, we found that expression level of HO-1 was not only correlated with Gleason grades, but also with aggressive pathologic features, such as tumor stage and PSA level. Importantly, we showed that miR-193a-5p overexpression further increased HO-1 expression level induced by docetaxel and attenuated PC3 cell apoptosis. Reversely, depletion of miR-193a-5p markedly reduced docetaxel-induced HO-1 expression and promoted PC3 cell apoptosis. These results suggest that miR-193a-5p mediates docetaxel regulation of HO-1 expression. We further used HO-1 inhibitor or HO-1 inducer to treat PC3 cells and determined the effects of HO-1 on docetaxel-induced PC3 cell apoptosis. Experimental results revealed that HO-1 upregulation increased the resistance of PC3 cells to docetaxel-induced apoptosis.

Previous studies have suggested that the anti-apoptotic genes Bcl-2 and Bcl-xL were markedly upregulated in paclitaxel-resistant hepatoma cell line, and Bcl-xL expression was enhanced by paclitaxel treatment [48]. In addition, Bcl-2 upregulation was observed in 30–60% of PC, as well as in nearly 100% of hormone-refractory PC [49]. Bax is a member of the Bcl-2 family and counteracted the anti-apoptotic roles of Bcl-2 [50]. Reagan-Shaw et al. reported that vitamin E and selenium induced apoptosis of the LNCaP, DU145 and PC3 cell lines through upregulating Bax, Bak and Bid, as well as through downregulating Bcl-2 [51]. These observations suggest that the anti-tumor effects of chemotherapeutic drugs occur through their regulation of the Bcl-2 signaling pathway. But there are only few evidences to show the relationship between HO-1, Bcl-2 and Bax in PC. In this study, we demonstrated for the first time that there is a positive correlation between Bcl-2 and HO-1 mRNAs but a negative correlation between Bax and HO-1 mRNAs in PC3 cells. Based on the fact that docetaxel combined with HO-1 inhibitor repressed Bcl-2 expression and enhanced Bax expression, whereas docetaxel combined with HO-1 inducer increased Bcl-2 and reduced Bax expression, it can be concluded that HO-1 upregulation leads to resistance of PC3 cells to docetaxel-induced apoptosis by increasing Bcl-2 and decreasing Bax expression.

There is no binding site of miR-193a-5p in the 3′-UTR of HO-1 gene. Thus, we thought that HO-1 may not be a direct target of miR-193a-5p. Because the HO-1 promoter has multiple copies of the antioxidant-response element (ARE) [35], and these elements can bind with the transcription factor Nrf2 [21] and the transcriptional repressor Bach1 [22], we determined whether miR-193a-5p suppresses the expression of transcription factors which regulate HO-1 expression. As expected,transfection of PC3 cells with miR-193a-5p mimic significantly decreased the expression of Bach2, but not Nrf2, Bach1 or Hif1α. Transfection of miR-193a-5p mimic combined with docetaxel treatment further attenuated Bach2 expression. Further, Bach2 3′-UTR-luciferase reporter assay revealed that miR-193a-5p downregulated Bach2 expression by directly targeting the miR-193a-5p-binding site in the Bach2 3′-UTR. Moreover, we found that Bach2 expression was significantly lower in PC than in BPH tissues, and expression level of miR-193a-5p closely correlated with Bach2 level in human PC tissues. A recent study revealed that Bach2 functioned in a variety of cellular lineages that can either promote or suppress immune responses against tumors [52]. Consistent with this, we showed that Bach2 induced PC cell apoptosis through repressing HO-1 expression. Notably, we found that a modest increase of HO-1 induced by docetaxel facilitated docetaxel-induced cell apoptosis. However, docetaxel-induced miR-193a-5p upregulation, which in turn inhibits Bach2 expression and thus enhances HO-1 expression, partly counteracts docetaxel-induced apoptosis. Therefore, silencing of miR-193a-5p can increase the sensitization of PC cells to docetaxel-induced apoptosis.

## Conclusions

In summary, we found that miR-193a-5p upregulation attenuated PC cell sensitivity to docetaxel-induced apoptosis through relieving Bach2 repression of HO-1 transcription. Silencing of miR-193a-5p or blockade of the miR-193a-5p-Bach2-HO-1 pathway may be a novel therapeutic approach for castration-resistant PC.

## Additional files

Additional file 1: Table S1. Patient and tumor characteristics. (TIFF 7 kb)

Additional file 2: Table S3. Sequence of primers or RNA. (TIFF 10 kb)

Additional file 3: Figure S1. Quantitative analysis of Fig. 1b. Data are expressed as mean ± SEM of fluorescence intensity in FISH assay (BPH, *n* = 33; PC, *n* = 24). ***P < 0.001 vs. BPH. (TIFF 25 kb)

Additional file 4: Figure S2. miR-193a-5p regulated the expression of HO-1 but not p22phox and p47phox in PC3 cell. Quantitative analysis of Fig. 3a. Data are expressed as mean ± SEM from three independent experiments. ***P < 0.001 vs. their corresponding control. (TIFF 309 kb)

Additional file 5: Figure S3. Quantitative analysis of Fig. 3b. Data are expressed as mean ± SEM of HO-1-positive cells in different stages of PC. 1, Benign prostatic hyperplasia (BPH); 2, Gleason grade 2; 3, Gleason grade 4; 4, Gleason grade 5 PC. *P < 0.05, **P < 0.01, ***P < 0.001 vs. their corresponding control. (TIFF 327 kb)

Additional file 6: Table S2. Association of HO-1 immunohistochemical expression with clinicopathological characteristic of prostatic carcinomas. (TIFF 311 kb)

Additional file 7: Figure S4. Quantitative analysis of Fig. 3f (A) and I (B). Data are expressed as mean ± SEM from three independent experiments. *P < 0.05, **P < 0.01, ***P < 0.001 vs. their corresponding control. (TIFF 368 kb)

Additional file 8: Figure S5. Quantitative analysis of Fig. 4a (A) and C (B). Data are expressed as mean ± SEM of the positive cells in different stages of PC. 1, Benign prostatic hyperplasia (BPH); 2, Gleason grade 2; 3, Gleason grade 4; 4, Gleason grade 5 PC. *P < 0.05, **P < 0.01, ***P < 0.001 vs. their corresponding control. (TIFF 339 kb)

Additional file 9: Figure S6. miR-193a-5p mediated Doc regulation of Bach2 expression. The mRNA of potential transcription factors for HO-1 was detected by qRT-PCR in PC3 cells treated with or without Doc after miR-193a-5p mimic or control mimic transfection. *P < 0.05 vs. their corresponding control. (TIFF 325 kb)

Additional file 10: Figure S7. miR-193a-5p did not affect expression of Maf in PC3 cells. A, PC3 cells were transfected with anti-miR-193a-5p, anti-miR-Ctl, miR-193a-5p mimic or mimic Ctl for 24 h, and Maf mRNA was detected by qRT-PCR. B, PC3 cells were transfected with the indicated RNA constructs and Maf protein was detected by Western blotting. C, Quantitative analysis of Maf protein. Data are expressed as mean ± SEM from three independent experiments. (TIFF 425 kb)

Additional file 11: Figure S8. Quantitative analysis of Fig. 8d. Data are expressed as mean ± SEM from three independent experiments. *P < 0.05, **P < 0.01 vs. their corresponding control. (TIFF 344 kb)

Additional file 12: Figure S9. Quantitative analysis of Fig. 8e. The number of TUNEL-positive cells of three independent experiments. *P < 0.01 vs. their corresponding control. (TIFF 319 kb)

## Abbreviations

ADT: Androgen Deprivation Therapy
ALL: Acute Lymphoblastic Leukemia
AP-1: Activator Protein-1
Bach1: BTB and CNC homolog 1
Bach2: BTB and CNC homolog 2
Bax: Apoptosis regulator BAX
BCL-2: Apoptosis regulator Bcl-2
CML: Chronic Myeloid Leukemia
Doc: Docetaxel
HO-1: Heme oxygenase-1
miRNAs: microRNAs
Nrf2: Nuclear factor erythroid 2-related factor 2
PC: Prostate Cancer
qPCR: Quantitative Polymerase Chain Reaction
TURP: Transurethral Resection of the Prostate
UTR: Untranslated Region

## References

[1] Wetherill YB, Hess-Wilson JK, Comstock CE, Shah SA, Buncher CR, Sallans L, Limbach PA, Schwemberger S, Babcock GF, Knudsen KE, Bisphenol A (2006). Facilitates bypass of androgen ablation therapy in prostate cancer. Mol Cancer Ther.

[2] Dehm SM, Tindall DJ (2006). Molecular regulation of androgen action in prostate cancer. J Cell Biochem.

[3] Eder IE, Haag P, Bartsch G, Klocker H (2005). Targeting the androgen receptor in hormone-refractory prostate cancer--new concepts. Future Oncol.

[4] Matsumoto A, Inoue A, Yokoi S, Nozumi K, Miyazaki K, Hosoki S, Nagata M, Yamaguchi K (2009). Evaluation of docetaxel plus estramustine in the treatment of patients with hormone-refractory prostate cancer. Int J Urol.

[5] Francini E, Sweeney CJ (2016). Docetaxel activity in the era of life-prolonging hormonal therapies for metastatic castration-resistant prostate cancer. Eur Urol.

[6] Puhr M, Hoefer J, Schafer G, Erb HH, SJ O, Klocker H, Heidegger I, Neuwirt H, Culig Z (2012). Epithelial-to-mesenchymal transition leads to docetaxel resistance in prostate cancer and is mediated by reduced expression of miR-200c and miR-205. Am J Pathol.

[7] Ye QF, Zhang YC, Peng XQ, Long Z, Ming YZ, He LY (2012). Silencing Notch-1 induces apoptosis and increases the chemosensitivity of prostate cancer cells to docetaxel through Bcl-2 and Bax. Oncol Lett.

[8] Nie CJ, Li YH, Zhang XH, Wang ZP, Jiang W, Zhang Y, Yin WN, Zhang Y, Shi HJ, Liu Y (2016). SUMOylation of KLF4 acts as a switch in transcriptional programs that control VSMC proliferation. Exp Cell Res.

[9] Wen D, Peng Y, Lin F, Singh RK, Mahato RI (2017). Micellar delivery of miR-34a modulator Rubone and paclitaxel in resistant prostate cancer. Cancer Res.

[10] Shi XB, Ma AH, Xue L, Li M, Nguyen HG, Yang JC, Tepper CG, Gandour-Edwards R, Evans CP, Kung HJ, deVere White RW (2015). miR-124 and androgen receptor signaling inhibitors repress prostate cancer growth by downregulating androgen receptor splice variants, EZH2, and Src. Cancer Res.

[11] Pennati M, Lopergolo A, Profumo V, De Cesare M, Sbarra S, Valdagni R, Zaffaroni N, Gandellini P, Folini M (2014). miR-205 impairs the autophagic flux and enhances cisplatin cytotoxicity in castration-resistant prostate cancer cells. Biochem Pharmacol.

[12] Shi GH, Ye DW, Yao XD, Zhang SL, Dai B, Zhang HL, Shen YJ, Zhu Y, Zhu YP, Xiao WJ, Ma CG (2010). Involvement of microRNA-21 in mediating chemo-resistance to docetaxel in androgen-independent prostate cancer PC3 cells. Acta Pharmacol Sin.

[13] Yang Y, Zhou L, Lu L, Wang L, Li X, Jiang P, Chan LK, Zhang T, Yu J, Kwong J (2013). A novel miR-193a-5p-YY1-APC regulatory axis in human endometrioid endometrial adenocarcinoma. Oncogene.

[14] Yu T, Li J, Yan M, Liu L, Lin H, Zhao F, Sun L, Zhang Y, Cui Y, Zhang F (2015). MicroRNA-193a-3p and -5p suppress the metastasis of human non-small-cell lung cancer by downregulating the ERBB4/PIK3R3/mTOR/S6K2 signaling pathway. Oncogene.

[15] Zhou J, Duan H, Xie Y, Ning Y, Zhang X, Hui N, Wang C, Zhang J, Zhou J (2016). MiR-193a-5p targets the coding region of AP-2alpha mRNA and induces cisplatin resistance in bladder cancers. J Cancer.

[16] Jacques C, Calleja LR, Baud'huin M, Quillard T, Heymann D, Lamoureux F, Ory B (2016). miRNA-193a-5p repression of p73 controls cisplatin chemoresistance in primary bone tumors. Oncotarget.

[17] Deng H, Lv L, Li Y, Zhang C, Meng F, Pu Y, Xiao J, Qian L, Zhao W, Liu Q (2014). miR-193a-3p regulates the multi-drug resistance of bladder cancer by targeting the LOXL4 gene and the oxidative stress pathway. Mol Cancer.

[18] Ryter SW, Alam J, Choi AM (2006). Heme oxygenase-1/carbon monoxide: from basic science to therapeutic applications. Physiol Rev.

[19] Li Y, Su J, DingZhang X, Zhang J, Yoshimoto M, Liu S, Bijian K, Gupta A, Squire JA, Alaoui Jamali MA, Bismar TA (2011). PTEN deletion and heme oxygenase-1 overexpression cooperate in prostate cancer progression and are associated with adverse clinical outcome. J Pathol.

[20] Sullivan R, Graham CH (2008). Chemosensitization of cancer by nitric oxide. Curr Pharm Des.

[21] Itoh K, Chiba T, Takahashi S, Ishii T, Igarashi K, Katoh Y, Oyake T, Hayashi N, Satoh K, Hatayama I (1997). An Nrf2/small Maf heterodimer mediates the induction of phase II detoxifying enzyme genes through antioxidant response elements. Biochem Biophys Res Commun.

[22] Ogawa K, Sun J, Taketani S, Nakajima O, Nishitani C, Sassa S, Hayashi N, Yamamoto M, Shibahara S, Fujita H, Igarashi K (2001). Heme mediates derepression of Maf recognition element through direct binding to transcription repressor Bach1. EMBO J.

[23] Kietzmann T, Samoylenko A, Immenschuh S (2003). Transcriptional regulation of heme oxygenase-1 gene expression by MAP kinases of the JNK and p38 pathways in primary cultures of rat hepatocytes. J Biol Chem.

[24] Zhang S, Yang X, Luo J, Ge X, Sun W, Zhu H, Zhang W, Cao J, Hou Y (2014). PPARalpha activation sensitizes cancer cells to epigallocatechin-3-gallate (EGCG) treatment via suppressing heme oxygenase-1. Nutr Cancer.

[25] Yoshida C, Yoshida F, Sears DE, Hart SM, Ikebe D, Muto A, Basu S, Igarashi K, Melo JV (2007). Bcr-Abl signaling through the PI-3/S6 kinase pathway inhibits nuclear translocation of the transcription factor Bach2, which represses the antiapoptotic factor heme oxygenase-1. Blood.

[26] Swaminathan S, Huang C, Geng H, Chen Z, Harvey R, Kang H, Ng C, Titz B, Hurtz C, Sadiyah MF (2013). BACH2 mediates negative selection and p53-dependent tumor suppression at the pre-B cell receptor checkpoint. Nat Med.

[27] Wang X, Gao H, Ren L, Gu J, Zhang Y, Zhang Y (2014). Demethylation of the miR-146a promoter by 5-Aza-2′-deoxycytidine correlates with delayed progression of castration-resistant prostate cancer. BMC Cancer.

[28] Yang Z, Zheng B, Zhang Y, He M, Zhang XH, Ma D, Zhang RN, Wu XL, Wen JK (1852). miR-155-dependent regulation of mammalian sterile 20-like kinase 2 (MST2) coordinates inflammation, oxidative stress and proliferation in vascular smooth muscle cells. Biochim Biophys Acta.

[29] Seo JY, Pyo E, An JP, Kim J, Sung SH, Oh WK (2017). Andrographolide activates Keap1/Nrf2/ARE/HO-1 pathway in HT22 cells and suppresses microglial activation by Abeta42 through Nrf2-related inflammatory response. Mediat Inflamm.

[30] Lewis BP, Burge CB, Bartel DP (2005). Conserved seed pairing, often flanked by adenosines, indicates that thousands of human genes are microRNA targets. Cell.

[31] Betel D, Koppal A, Agius P, Sander C, Leslie C (2010). Comprehensive modeling of microRNA targets predicts functional non-conserved and non-canonical sites. Genome Biol.

[32] Kirkegaard T, Edwards J, Tovey S, McGlynn LM, Krishna SN, Mukherjee R, Tam L, Munro AF, Dunne B, Bartlett JM (2006). Observer variation in immunohistochemical analysis of protein expression, time for a change?. Histopathology.

[33] Walter BA, Valera VA, Pinto PA, Merino MJ (2013). Comprehensive microRNA profiling of prostate cancer. J Cancer.

[34] Heinemann L, Simpson GR, Boxall A, Kottke T, Relph KL, Vile R, Melcher A, Prestwich R, Harrington KJ, Morgan R, Pandha HS (2011). Synergistic effects of oncolytic reovirus and docetaxel chemotherapy in prostate cancer. BMC Cancer.

[35] Pulkkinen KH, Yla-Herttuala S, Levonen AL (2011). Heme oxygenase 1 is induced by miR-155 via reduced BACH1 translation in endothelial cells. Free Radic Biol Med.

[36] Sasaki S, Ito E, Toki T, Maekawa T, Kanezaki R, Umenai T, Muto A, Nagai H, Kinoshita T, Yamamoto M (2000). Cloning and expression of human B cell-specific transcription factor BACH2 mapped to chromosome 6q15. Oncogene.

[37] Patterson SG, Wei S, Chen X, Sallman DA, Gilvary DL, Zhong B, Pow-Sang J, Yeatman T, Djeu JY (2006). Novel role of Stat1 in the development of docetaxel resistance in prostate tumor cells. Oncogene.

[38] Xie Y, Xu K, Linn DE, Yang X, Guo Z, Shimelis H, Nakanishi T, Ross DD, Chen H, Fazli L (2008). The 44-kDa Pim-1 kinase phosphorylates BCRP/ABCG2 and thereby promotes its multimerization and drug-resistant activity in human prostate cancer cells. J Biol Chem.

[39] Ploussard G, Terry S, Maille P, Allory Y, Sirab N, Kheuang L, Soyeux P, Nicolaiew N, Coppolani E, Paule B (2010). Class III beta-tubulin expression predicts prostate tumor aggressiveness and patient response to docetaxel-based chemotherapy. Cancer Res.

[40] Cao Q, Mao ZD, Shi YJ, Chen Y, Sun Y, Zhang Q, Song L, Peng LP (2016). MicroRNA-7 inhibits cell proliferation, migration and invasion in human non-small cell lung cancer cells by targeting FAK through ERK/MAPK signaling pathway. Oncotarget.

[41] Leivonen SK, Sahlberg KK, Makela R, Due EU, Kallioniemi O, Borresen-Dale AL, Perala M (2014). High-throughput screens identify microRNAs essential for HER2 positive breast cancer cell growth. Mol Oncol.

[42] Huang C, CK L, MC T, Chang JH, Chen YJ, YH T, Huang HC (2016). Polyphenol-rich Avicennia Marina leaf extracts induce apoptosis in human breast and liver cancer cells and in a nude mouse xenograft model. Oncotarget.

[43] Rajput S, Mandal M (2012). Antitumor promoting potential of selected phytochemicals derived from spices: a review. Eur J Cancer Prev.

[44] Alaoui-Jamali MA, Bismar TA, Gupta A, Szarek WA, Su J, Song W, Xu Y, Xu B, Liu G, Vlahakis JZ (2009). A novel experimental heme oxygenase-1-targeted therapy for hormone-refractory prostate cancer. Cancer Res.

[45] Busserolles J, Megias J, Terencio MC, Alcaraz MJ (2006). Heme oxygenase-1 inhibits apoptosis in Caco-2 cells via activation of Akt pathway. Int J Biochem Cell Biol.

[46] Lee SS, Yang SF, Tsai CH, Chou MC, Chou MY, Chang YC (2008). Upregulation of heme oxygenase-1 expression in areca-quid-chewing-associated oral squamous cell carcinoma. J Formos Med Assoc.

[47] Sacca P, Meiss R, Casas G, Mazza O, Calvo JC, Navone N, Vazquez E (2007). Nuclear translocation of haeme oxygenase-1 is associated to prostate cancer. Br J Cancer.

[48] Chun E, Lee KY (2004). Bcl-2 and Bcl-xL are important for the induction of paclitaxel resistance in human hepatocellular carcinoma cells. Biochem Biophys Res Commun.

[49] Grad JM, Zeng XR, Boise LH (2000). Regulation of Bcl-xL: a little bit of this and a little bit of STAT. Curr Opin Oncol.

[50] Yoshino T, Shiina H, Urakami S, Kikuno N, Yoneda T, Shigeno K, Igawa M (2006). Bcl-2 expression as a predictive marker of hormone-refractory prostate cancer treated with taxane-based chemotherapy. Clin Cancer Res.

[51] Reagan-Shaw S, Nihal M, Ahsan H, Mukhtar H, Ahmad N (2008). Combination of vitamin E and selenium causes an induction of apoptosis of human prostate cancer cells by enhancing Bax/Bcl-2 ratio. Prostate.

[52] Roychoudhuri R, Eil RL, Clever D, Klebanoff CA, Sukumar M, Grant FM, Yu Z, Mehta G, Liu H, Jin P (2016). The transcription factor BACH2 promotes tumor immunosuppression. J Clin Invest.

