# Supplementary figures and images for: Silencing of miR-193a-5p increases the chemosensitivity of prostate cancer cells to docetaxel
Source: J Exp Clin Cancer Res. 2017 Dec 8;36:178. doi: 10.1186/s13046-017-0649-3 (PMC5721613; doi:10.1186/s13046-017-0649-3)

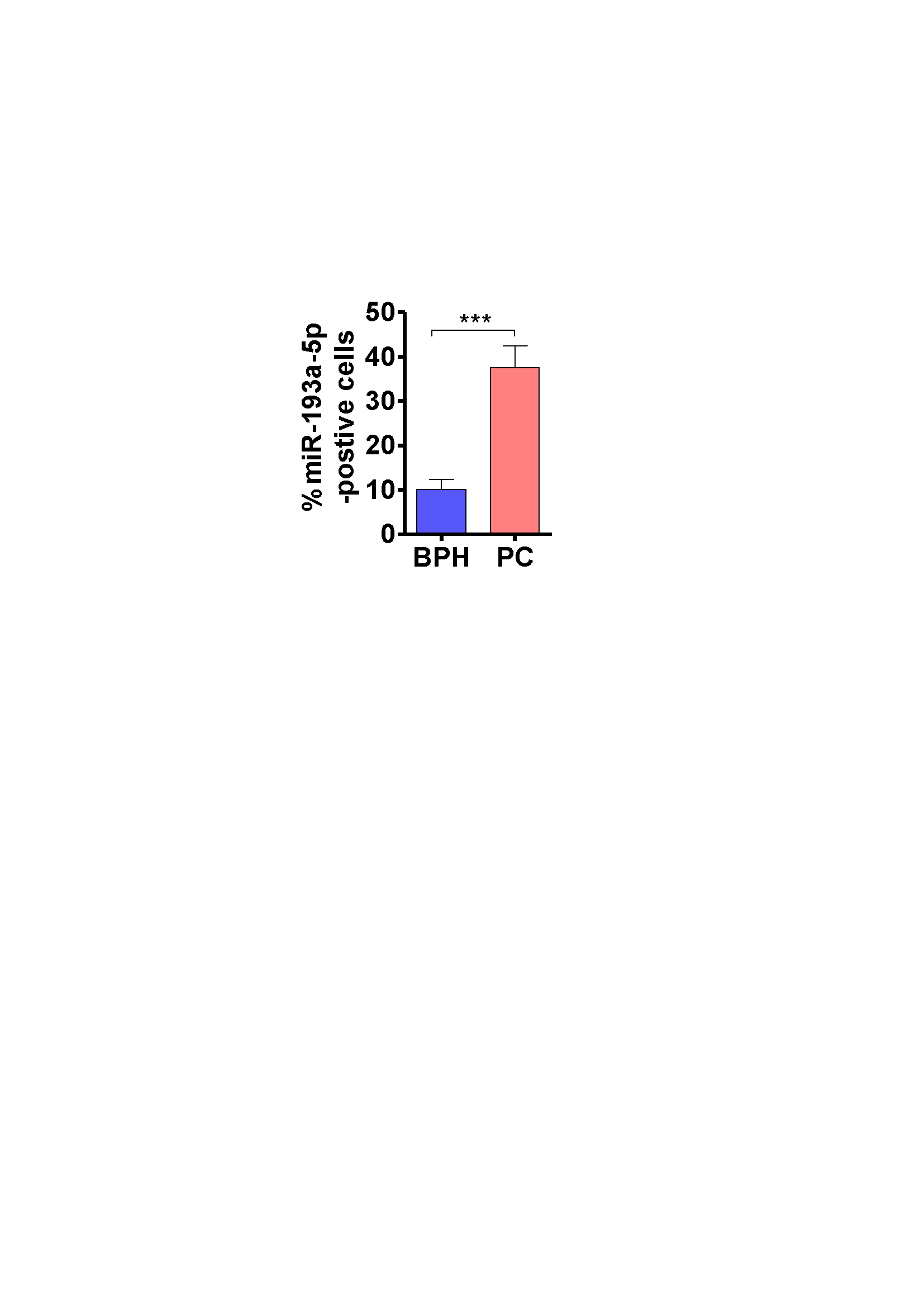

Supplement: Supplementary file 4 — miR-193a-5p regulated the expression of HO-1 but not p22phox and p47phox in PC3 cell. Quantitative analysis of Fig. 3a. Data are expressed as mean ± SEM from three independent experiments. ***P < 0.001 vs. their corresponding control. (TIFF 309 kb) [file 13046_2017_649_MOESM4_ESM.tiff]

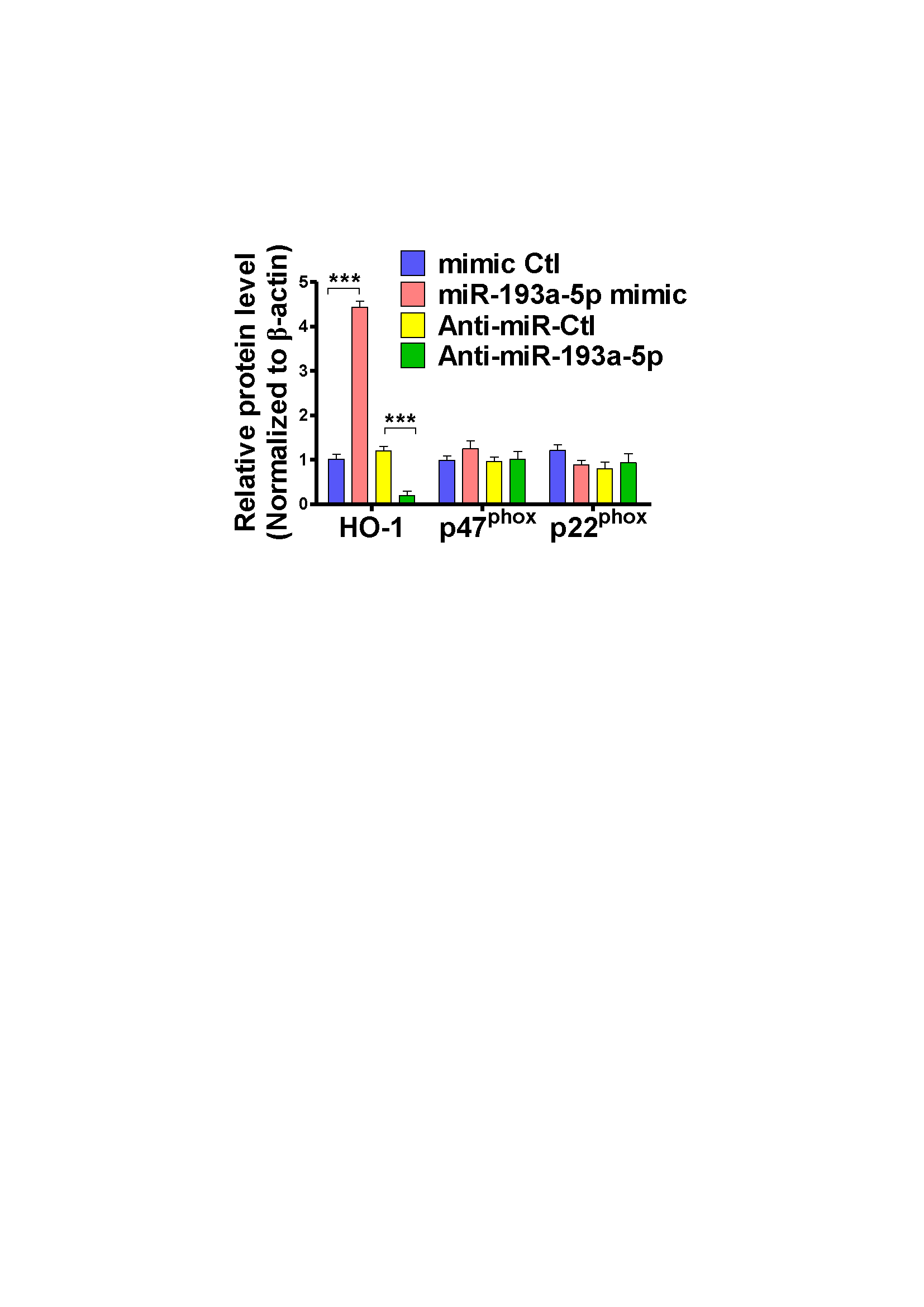

Supplement: Supplementary file 5 — Quantitative analysis of Fig. 3b. Data are expressed as mean ± SEM of HO-1-positive cells in different stages of PC. 1, Benign prostatic hyperplasia (BPH); 2, Gleason grade 2; 3, Gleason grade 4; 4, Gleason grade 5 PC. *P < 0.05, **P < 0.01, ***P < 0.001 vs. their corresponding control. (TIFF 327 kb) [file 13046_2017_649_MOESM5_ESM.tiff]

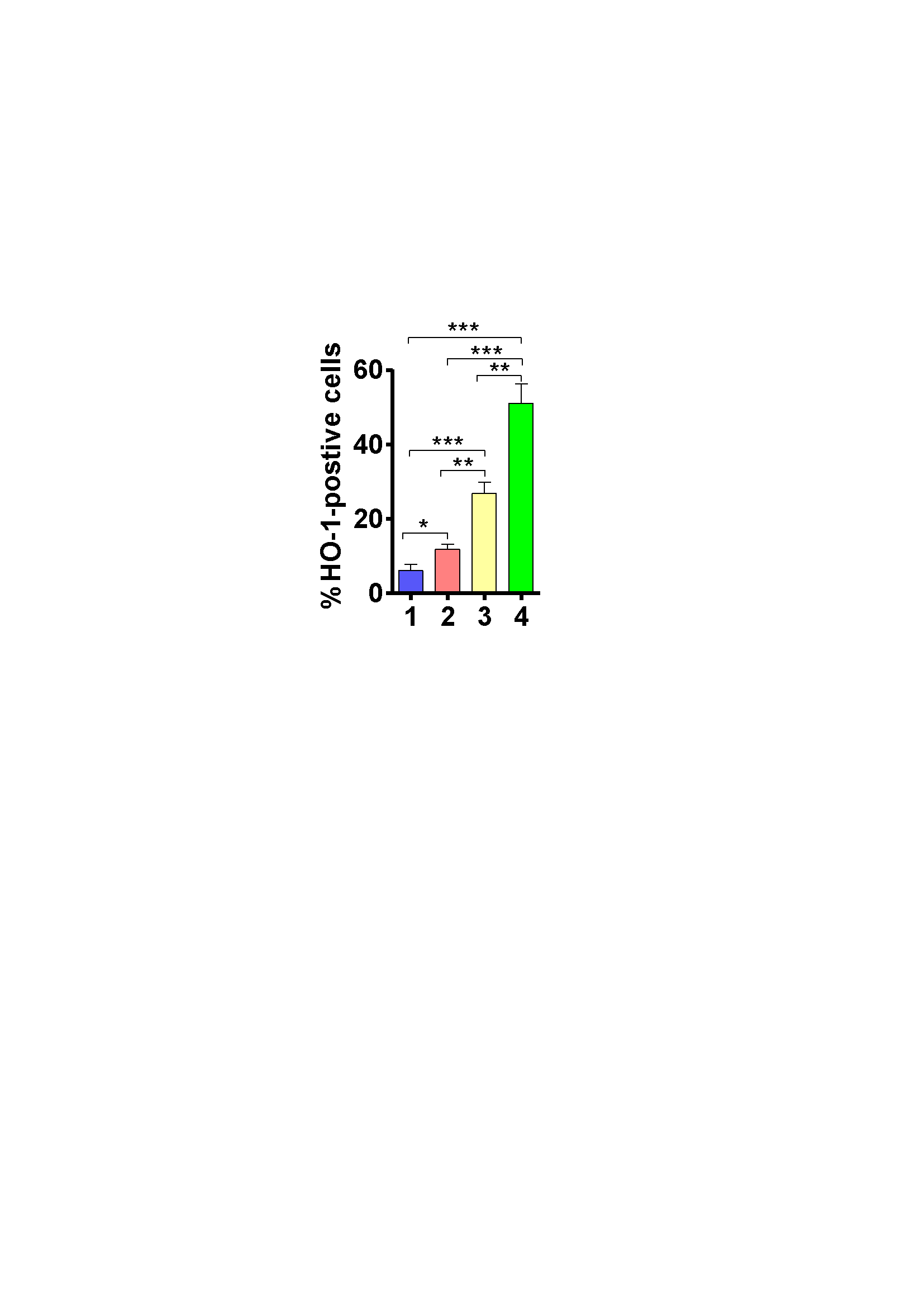

Supplement: Supplementary file 6 — Association of HO-1 immunohistochemical expression with clinicopathological characteristic of prostatic carcinomas. (TIFF 311 kb) [file 13046_2017_649_MOESM6_ESM.tiff]

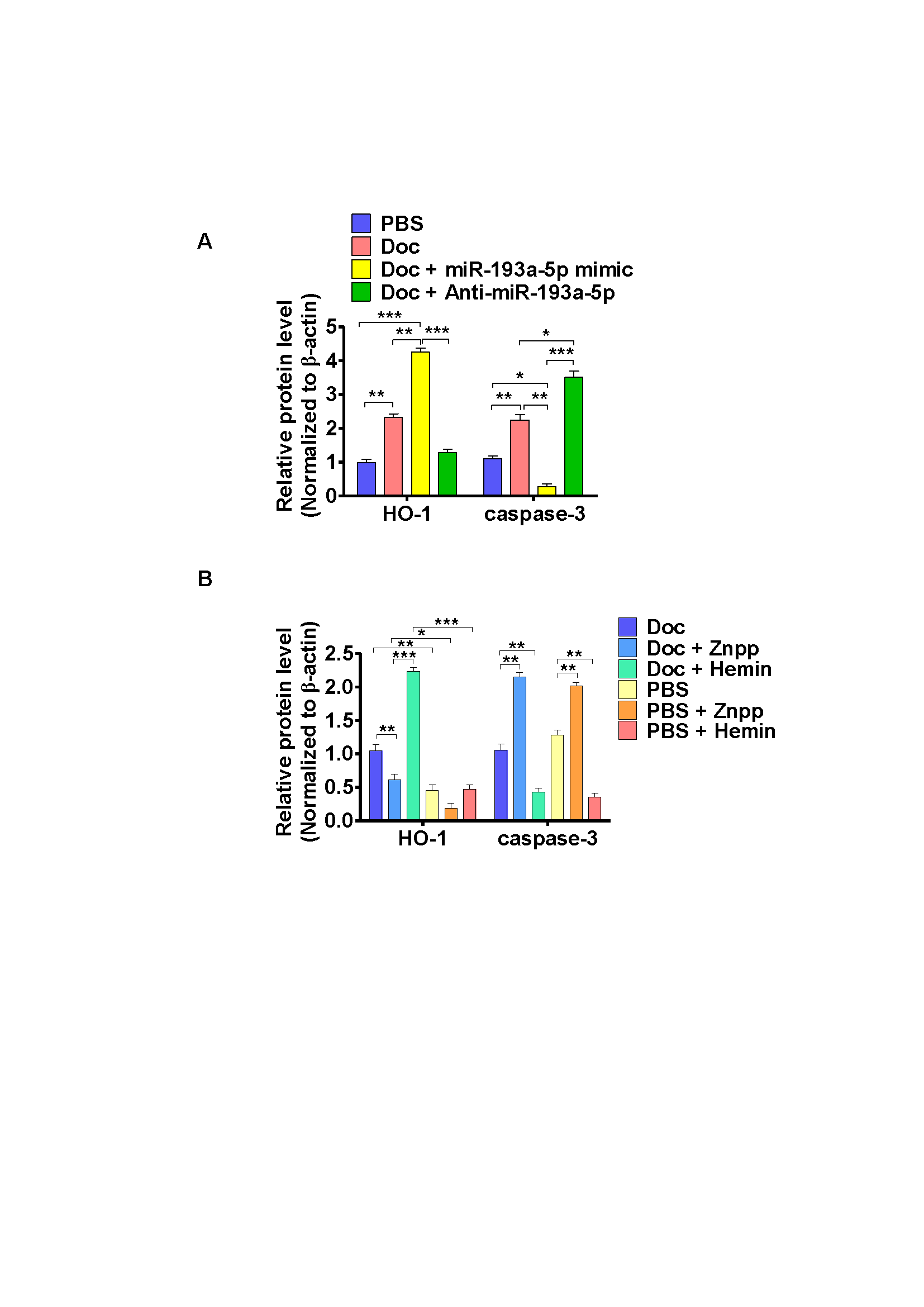

Supplement: Supplementary file 7 — Quantitative analysis of Fig. 3f (A) and I (B). Data are expressed as mean ± SEM from three independent experiments. *P < 0.05, **P < 0.01, ***P < 0.001 vs. their corresponding control. (TIFF 368 kb) [file 13046_2017_649_MOESM7_ESM.tiff]

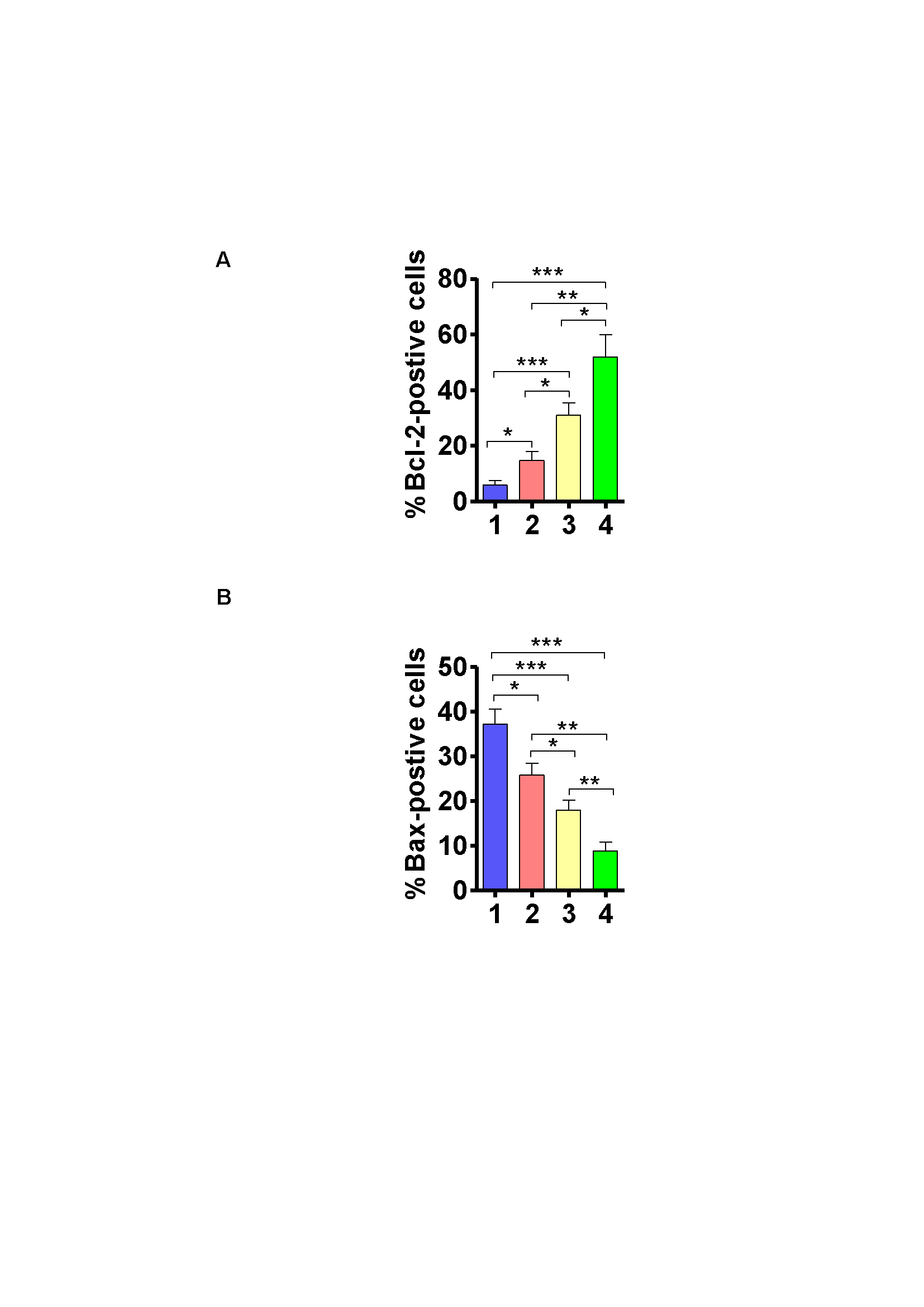

Supplement: Supplementary file 8 — Quantitative analysis of Fig. 4a (A) and C (B). Data are expressed as mean ± SEM of the positive cells in different stages of PC. 1, Benign prostatic hyperplasia (BPH); 2, Gleason grade 2; 3, Gleason grade 4; 4, Gleason grade 5 PC. *P < 0.05, **P < 0.01, ***P < 0.001 vs. their corresponding control. (TIFF 339 kb) [file 13046_2017_649_MOESM8_ESM.tiff]

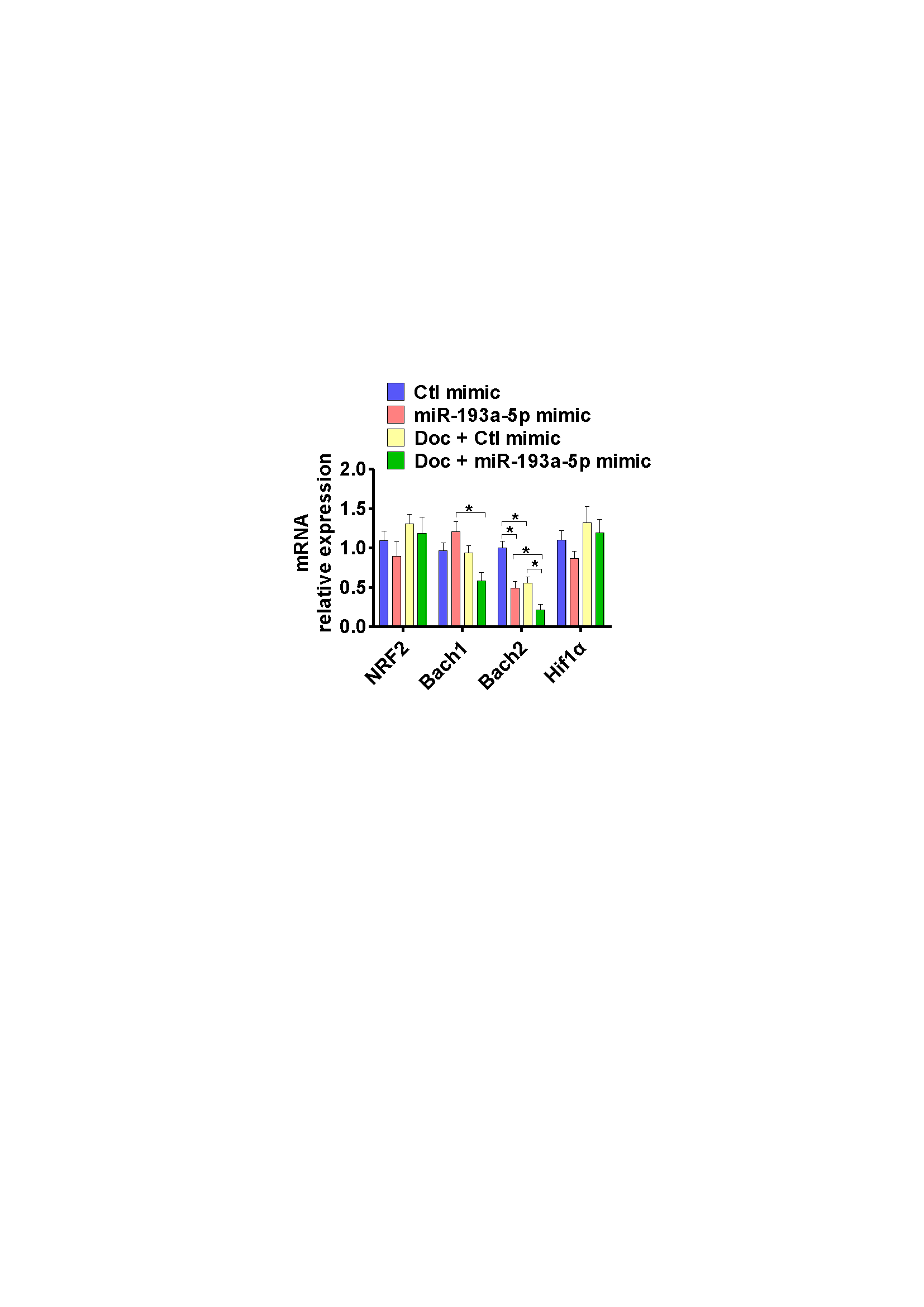

Supplement: Supplementary file 9 — miR-193a-5p mediated Doc regulation of Bach2 expression. The mRNA of potential transcription factors for HO-1 was detected by qRT-PCR in PC3 cells treated with or without Doc after miR-193a-5p mimic or control mimic transfection. *P < 0.05 vs. their corresponding control. (TIFF 325 kb) [file 13046_2017_649_MOESM9_ESM.tiff]

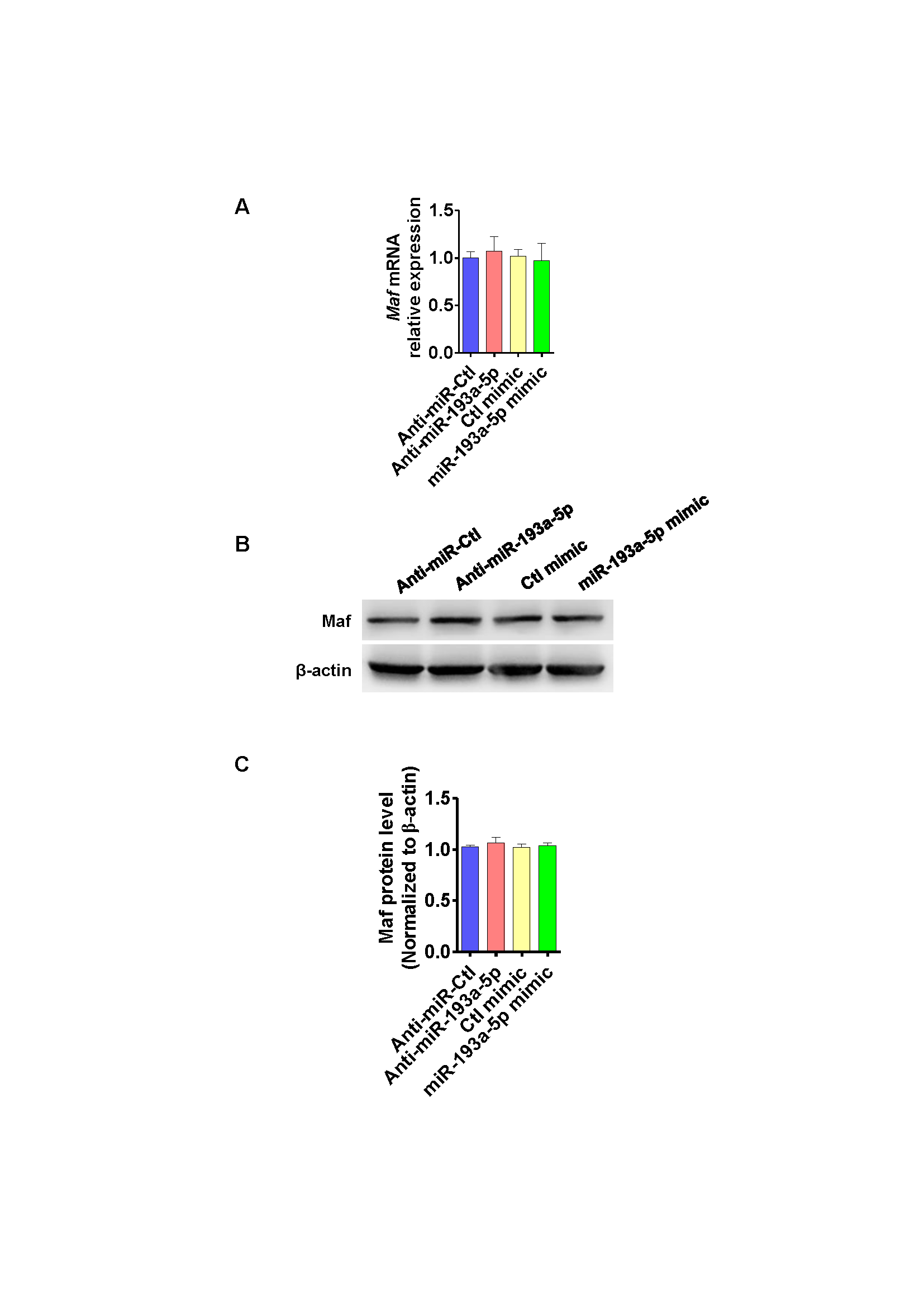

Supplement: Supplementary file 10 — miR-193a-5p did not affect expression of Maf in PC3 cells. A, PC3 cells were transfected with anti-miR-193a-5p, anti-miR-Ctl, miR-193a-5p mimic or mimic Ctl for 24 h, and Maf mRNA was detected by qRT-PCR. B, PC3 cells were transfected with the indicated RNA constructs and Maf protein was detected by Western blotting. C, Quantitative analysis of Maf protein. Data are expressed as mean ± SEM from three independent experiments. (TIFF 425 kb) [file 13046_2017_649_MOESM10_ESM.tiff]

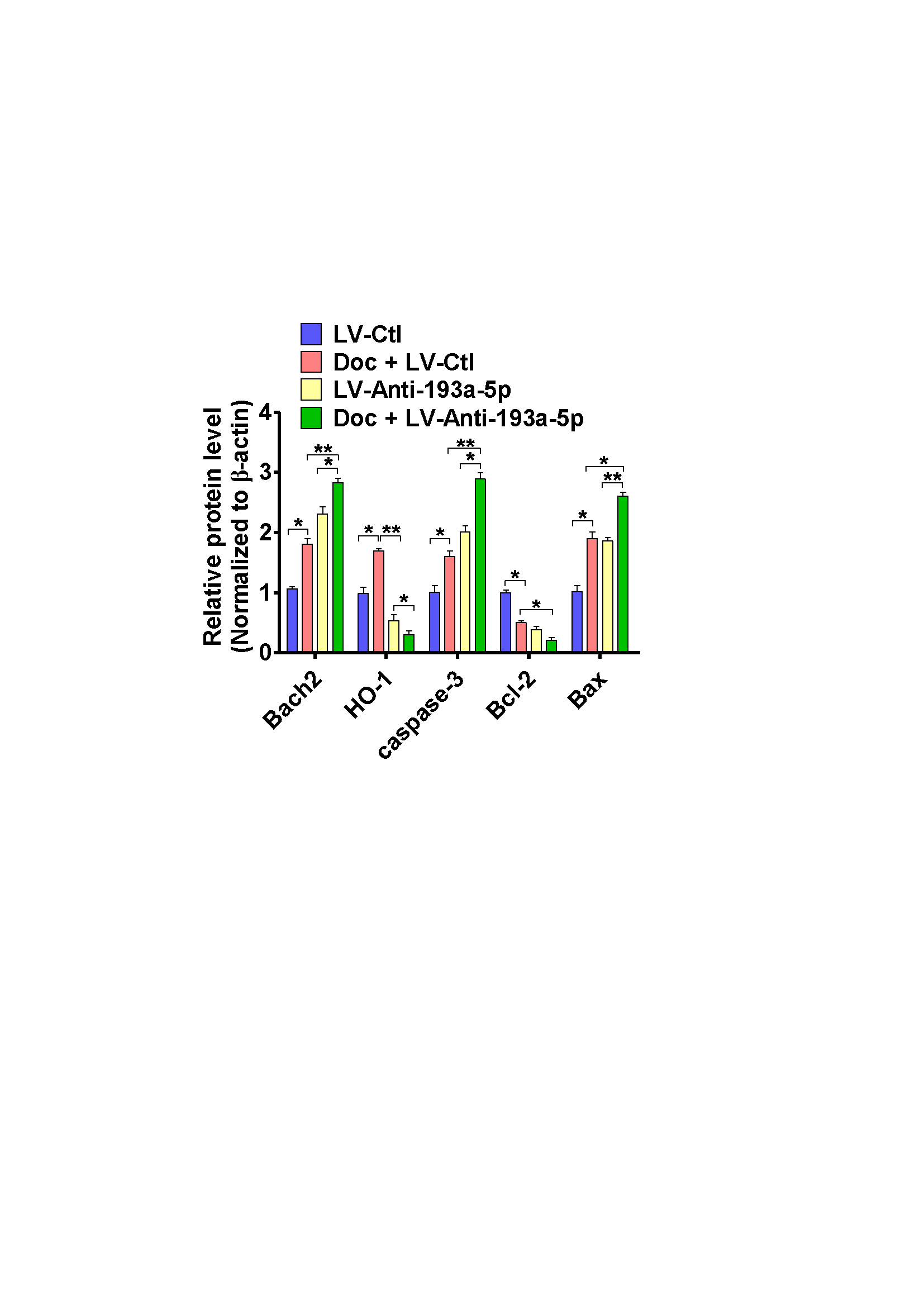

Supplement: Supplementary file 11 — Quantitative analysis of Fig. 8d. Data are expressed as mean ± SEM from three independent experiments. *P < 0.05, **P < 0.01 vs. their corresponding control. (TIFF 344 kb) [file 13046_2017_649_MOESM11_ESM.tiff]

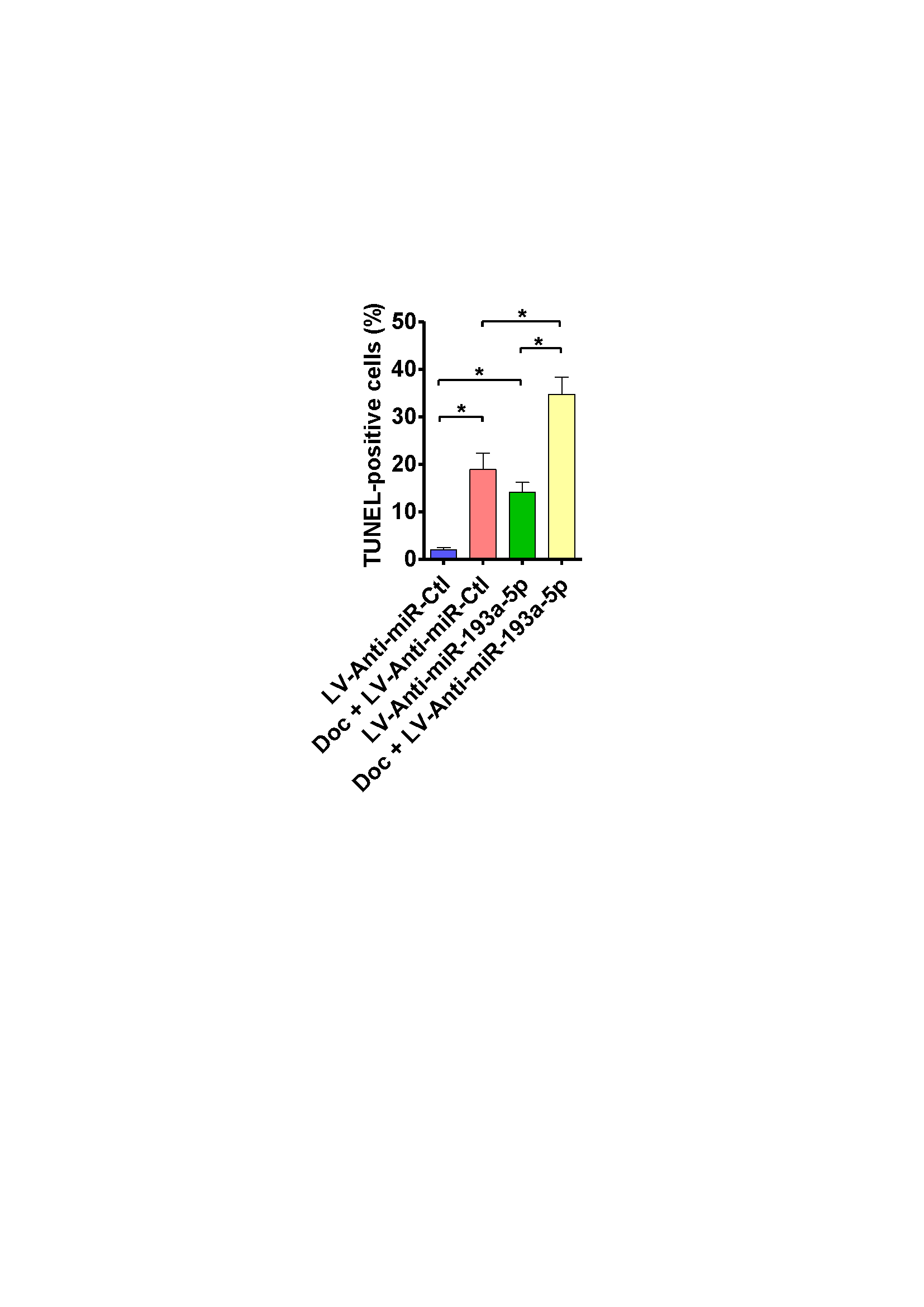

Supplement: Supplementary file 12 — Quantitative analysis of Fig. 8e. The number of TUNEL-positive cells of three independent experiments. *P < 0.01 vs. their corresponding control. (TIFF 319 kb) [file 13046_2017_649_MOESM12_ESM.tiff]
